# Utilization and quality: How the quality of care influences demand for obstetric care in Nigeria

**DOI:** 10.1371/journal.pone.0211500

**Published:** 2019-02-07

**Authors:** Evan D. Peet, Edward N. Okeke

**Affiliations:** 1 RAND Corporation, Pittsburgh, PA, United States of America; 2 RAND Corporation, Arlington, VA, United States of America; University of Ghana, GHANA

## Abstract

This paper examines the association between health facility quality, subjective perceptions, and utilization of obstetric care. We draw on unique survey data from Nigeria describing the quality of care at rural primary health care facilities and the utilization of obstetric care by households in the service areas of these facilities. Constructing a quality index using the detailed survey data, we show that facility quality is positively related to perceptions of quality and utilization. Disaggregating quality into structural, process and outcome dimensions, we find a consistently strong relationship only between utilization and structural measures of quality. The results suggest that efforts to improve quality may involve a trade-off between investing in dimensions that are more easily observed by households, which will influence utilization, and investing in dimensions that are more closely related to outcomes.

## Introduction

Despite global reductions in mortality and increasing life expectancies, large gaps remain, particularly in maternal and newborn health [1]. In 2015, 303,000 women died during and following pregnancy and childbirth, 4.2 million infants died within the first year of life, and 2.6 million babies were stillborn [2–4]. Nearly all maternal and child deaths occur in low- and middle-income countries: women living in poor countries are nearly 23 times more likely to die from pregnancy and childbirth-related complications than their counterparts living in developed countries [5]. Pregnancy-related morbidity and mortality has serious economic and social consequences, with estimates of the global productivity losses attributable to maternal and newborn deaths reaching approximately US $15 billion annually [6].

Proper obstetric care is considered to be a crucial determinant of birth outcomes [7–8], but remains underutilized in many low and middle-income countries. In the least developed countries that account for the majority of global maternal and newborn deaths, over 50% of births take place at home without formal assistance [5, 7, 9]. Concerns about underutilization of care have led to various policy interventions designed to stimulate demand. Many of these policies have focused on relaxing demand-side constraints such as access and cost [10–12]. However, even when demand-side factors are addressed, many women continue to choose to give birth at home [13–14]. Research suggests that traditional attendants are often viewed as being as good as, or in some cases even better than, formally trained birth attendants [13, 15], and there is evidence that formal care is not always perceived as being associated with better outcomes [16–17]. Despite being a global health priority, demand-side efforts have not yielded the expected gains in utilization in many settings. One potential reason is that utilization is also constrained on the supply side [18]. Consequently, it is becoming increasingly obvious that efforts to incentivize utilization must also address supply-side constraints.

Poor quality of care is believed to be an important supply-side constraint [19–20], and there is evidence that poor quality of care can counteract the effects of demand-side interventions [21]. Low quality may not only constrain improvement in outcomes, it may also depress demand [22–23]. The latter is of particular interest. Understanding the relationship between quality and utilization, however, is challenging in part because quality is multi-dimensional [24]. It encompasses ‘structural’ factors such as availability of resources in health facilities, ‘process’ factors such as quality of diagnostics, and ‘outcomes’—or the end result of care provided [24]. Conceptually it is not obvious that each of these dimensions will have the same effects on demand. Some aspects of quality are not easily observed (e.g., a provider’s true level of competence), or their importance may not be obvious to non-medically trained individuals. This suggests that more easily observed dimensions of quality (e.g., structural factors) may have greater effects on demand even when their correlations with outcomes might be weak [18]. Therefore, there may be an inherent policy tradeoff between the effects of an intervention on demand and its effects on outcomes. This necessitates a deeper understanding of how quality influences demand, and specifically, which dimensions are important.

Much of the existing evidence on quality and utilization is from qualitative studies and there are few rigorous quantitative studies [14–23, 25–28]. This paper attempts to fill this gap by quantitatively examining the relationship between health facility quality and demand for obstetric care. Importantly, we are able to disaggregate quality into its different dimensions and examine how each dimension correlates with demand. We also examine the extent to which perceptions of quality are correlated with objectively measured quality. Several studies have shown that perceptions are important determinants of utilization [29]. Understanding how this relates to objective quality is an important but understudied question [30].

## Materials and methods

### Ethics statement

Ethical approvals for the study were granted by the RAND Human Subjects Protection Committee and the Aminu Kano Teaching Hospital Research Ethics Committee in Nigeria. Each participant gave informed consent prior to participation in the study. The human subject data of this study were analyzed anonymously.

### Data

We draw on data from the Better Obstetrics in Rural Nigeria (BORN) study. This study measured availability and quality of obstetric care and birth outcomes in rural communities in Nigeria [31]. Trained research assistants collected extensive data on service availability and quality from primary health care facilities and also surveyed women with a recent birth living in the communities served by these facilities. The health facilities in the sample consisted of rural public primary health care facilities that provided obstetric services. Primary health care facilities are the point of entry for most patients into the health care system in Nigeria. Participating facilities were spread across 12 States covering all six geopolitical regions in Nigeria. In total, 362 primary health care facilities were surveyed [31].

Data collection took place between June 2014 and January 2015. In each health facility, extensive data were collected on service delivery, staffing and availability of supplies and equipment. The research assistant also observed and recorded the physical condition of the clinic. In addition, a randomly selected midwife (or other health worker if no midwife was available) was interviewed. Health workers were asked about their qualifications, the length of time they had been employed at the clinic, and satisfaction with various aspects of their work environment. Their clinical competence was also assessed using a combination of multiple-choice questions and clinical case studies.

Additionally, research assistants surveyed a random sample of approximately 20 households in each community where the facility was located. Since a comprehensive listing of households in each community was unavailable, we randomly generated 20 random GPS coordinates within each community using a GPS-enabled tablet and special software and selected the dwelling nearest this point for interview. If there was no eligible household within the dwelling, the interviewer visited the dwelling on either side until one was found. If there were multiple eligible households within the dwelling, one was randomly chosen for interview. Eligibility for participation was based on having a female member who was pregnant within the preceding five years. This eligibility criteria was adopted in line with the commonly accepted practice of using a five-year recall period as is used in the Demographic and Health Surveys. All such women within the household were interviewed. General information was collected about household characteristics and ownership of various household assets. An individual module collected demographic information as well as information on use of antenatal care, and place of delivery for prior births.

To account for the fact that facility characteristics are likely to change over time, we restrict our attention to the deliveries that occurred in the 12 months immediately preceding the health facility survey. Restricting the sample from deliveries that occurred within the five years preceding the health facility survey to those that occurred within 1 year reduced the number of observations from 8,902 to 2,140. The 2,140 deliveries comprise our analytic sample. Summary characteristics are reported in Table 1.

**Table 1:** Summary statistics of individual and household characteristics, perception of facility quality, and facility utilization.

	Obs.	Mean	Std. Dev.
**Individual Characteristics**			
Mother's age at birth	2,140	26.7	6.72
Mother's education			
None	2,140	29.3%	0.46
Koranic	2,140	10.0%	0.30
Some primary	2,140	17.5%	0.48
Some secondary	2,140	33.6%	0.47
Some tertiary	2,140	8.4%	0.28
Unknown	2,140	1.2%	0.14
Mother's Ethnicity			
Fulani	2,140	12.3%	0.34
Hausa	2,140	28.7%	0.46
Igbo	2,140	21.9%	0.39
Yoruba	2,140	16.1%	0.37
Other	2,140	20.8%	0.41
Mother is illiterate	2,140	46.6%	0.50
Mother believes facility birth unnecessary	2,140	14.9%	0.36
**Household Characteristics**			
Number of household assets	2,140	5.5	3.27
Health care Access and Costs			
Cost of delivery and drugs	2,140	3362.9	16823.39
Travel time in minutes to health facility	2,140	40.5	176.02
Cost of transportation to health facility (Naira)	2,140	198.9	930.03
Minutes to referral hospital	2,140	41.9	35.24
**Perception of Quality**			
Perception of Quality (1–4, 1 = poor, …, 4 = excellent)	2,140	2.8	0.70
**Utilization (Antenatal Care)**			
Antenatal care at any formal facility	2,140	84.7%	0.22
Antenatal care at the study facility	2,140	65.1%	0.48
Antenatal care at other public facility (including hospital)	2,140	13.6%	0.34
Antenatal care at private facility	2,140	6.0%	0.23
**Utilization (Facility Delivery)**			
Delivery at any formal facility	2,140	72.1%	0.45
Delivery at the study facility	2,140	51.0%	0.51
Delivery at other public facility (including hospital)	2,140	13.9%	0.35
Delivery at private facility	2,140	7.1%	0.26

Notes: The sample includes *N* = 2,140 deliveries that occurred in the 12 months immediately preceding the facility surveys. Study participants directly report each of the characteristics reported in the table, with the exception of literacy, which was assessed by asking respondents to read a simple sentence.

#### Key variables

The main dependent variable is utilization of obstetric care (antenatal and delivery care) in the primary health care facility. We define binary indicators for utilization of antenatal and delivery care at the primary health care facility. We also examine utilization of antenatal and delivery care at other public facilities, private facilities, and overall utilization of formal care. Our secondary dependent variable is perceptions of the primary health care facility quality. Women were asked to rate services in the facility as poor (1), average (2), good (3), or excellent (4). To analyze perceptions, the woman’s rating is used as the dependent variable.

For both the primary and secondary dependent variables, our main independent variable is care quality. Drawing on the Donabedian framework, we measure care quality along three dimensions: structure, process, and outcomes [24]. Using our rich survey data, we identify key measures in each of these domains. We conceptualize each of these measures as mapping loosely to a sub-domain of care quality. We use principal component analysis to extract an index for each sub-domain, each domain—structure, process, and outcomes—and to create an index of overall quality. Principal component analysis (PCA) is a procedure that generates orthogonal eigenvectors of a covariance matrix, and retains maximum variation [32]. While we acknowledge that there are limitations to assessing aggregated measures of quality generated by PCA, the objective of the study is to understand how quality, generally, influences utilization decisions rather than how one specific measure of quality influences utilization.

#### Analysis

Our objective is to relate variation in facility quality to utilization of obstetric care. We estimate the following using a linear probability model. We also verify that the results are robust to alternative specifications, including a logit model.

$$U_{i}=\alpha +\beta^{Q}Q_{i}+\gamma X_{i}+\varepsilon_{i}$$ (1)

*U*_*i*_ denotes the utilization outcome of interest for birth *i* and *Q*_*i*_ is the measure of quality. ***X***_***i***_ is a vector of covariates including individual and household characteristics such as maternal education, maternal literacy, mother’s age at child birth, mother’s ethnicity, mother’s belief in the necessity of a facility birth, household assets, cost of delivery and drugs, distance to facility in minutes, cost to reach facility, minutes to a referral hospital (each described in Table 1). Finally, *ε*_*i*_ describes the error term which is clustered at the facility-level. The coefficient *β*^*Q*^ describes the influence of overall facility quality on the outcome of interest. If *β*^*Q*^ = 0 then the care quality provided at the facility is unrelated to the outcome; in contrast, if $\beta^{Q}\neq 0$ then utilization of the facility is associated with the facility’s quality.

The next step in our analysis is to assess the effect of each of the domains of care quality–structure, process and outcomes–on utilization. We denote the structural index as $S_{i}$, the process index as $P_{i}$, and the outcomes index as $O_{i}$. We can then write the following model:

$$U_{i}=\alpha +\beta^{S}S_{i}+\beta^{P}P_{i}+\beta^{O}O_{i}+\gamma X_{i}+\varepsilon_{i}$$ (2)

$\beta^{S},\beta^{P}$, and $\beta^{O}$ therefore describe how structural quality, process quality, and outcome quality influence facility utilization. We also explore the contributions of each sub-domain (results are in the Appendix, S1 File).

Given that we exploit cross-sectional variation in facility quality, there is a concern that because higher-quality facilities are not randomly distributed, there may be a correlation between facility quality and household and individual characteristics that confound the relationship estimated by Eqs (1) and (2). To try to address this, we make use of propensity score-based methods. The idea is to compare similar women exposed to different levels of quality. Methods based on the propensity score have long been used for causal inference in observational studies; they are easy to use and can reduce the bias caused by non-random treatment assignment. There is evidence that in certain situations the propensity score method produces more reliable estimates of causal effects than other estimation methods [33–34]. Among the various propensity score methods developed for continuously valued treatments, we estimate the relationships described in Eqs (1) and (2) using the inverse second moment weighting method (ISMW) [35], and the weighted regression (WTRG) [36]. The ISMW approach is an extension of the inverse probability treatment weight approach using a marginal structural model that applies weights derived from propensity scores and generates a pseudo-population that mimics the properties of the overall population to correct distortions that arise from selection [37]. The WTRG method is a doubly robust estimator that uses inverse probability weights designed to give consistent estimates in a model for outcome prediction [36]. Further discussion is provided in the Appendix, S1 File. These propensity score-based estimators serve as a robustness checks for our main estimates generated using ordinary least squares. If the estimates are similar, the results are suggestive of a causal relationship. The results of these analyses are in the Appendix, S1 File.

## Results

### Summary characteristics

Table 1 reports means and standard deviations for the birth sample. Slightly over half the mothers received at least some secondary education and are literate. The mean age of mothers at the birth of the child is 26.7 years. 15% of the mothers believe that giving birth at a formal facility is unnecessary. For 65% of births, women utilized antenatal care at the primary health care facility. 51% of deliveries also occurred in the primary health facility (28% occurred at home). Table 2 describes the components of the quality indices and the mean and standard deviation of the variables comprising each index. Each component of the three indices of quality is measured at the facility level except for measures of antenatal and postnatal care quality, which come from the women’s survey. Neonatal, obstetric, and maternal outcomes are given as rates relative to the number of deliveries at the facility during the 12 months preceding the survey. The correlations between the overall index, the domain indices, the sub-domain indices and the indicators are shown in the Appendix, S1 File. For ease of interpretation, we standardize each index.

**Table 2:** Summary statistics of index components.

Indices and Sub-indices	Basic Measures	Obs.	Mean	Std. Dev.
**Structure**				
Size:	Number of beds	354	11.94	9.31
	Number of staff	362	9.34	6.10
Providers:	Number of doctors	362	0.30	1.58
	Number of nurses	355	0.44	1.20
	Number of midwives	355	2.85	1.80
	Percent with 24hr/7day provider availability	355	74%	0.44
	Percent with 24hr/7day delivery services	355	87%	0.34
Equipment:	Percent with supply of medicines	354	82%	0.39
	Percent with adult weight scale	354	94%	0.24
	Percent with baby weight scale	354	93%	0.25
	Percent with delivery bed	354	92%	0.27
	Percent with midwifery kit	354	61%	0.49
	Percent with delivery kit	354	66%	0.47
	Percent with incubator	354	3%	0.17
Clinical infrastructure:	Percent with laboratory	355	55%	0.50
	Percent with pharmacy	354	58%	0.49
	Percent with functional ambulance	354	10%	0.30
General infrastructure:	Percent with electricity grid connection	354	59%	0.49
	Percent with functional generator	354	46%	0.50
	Percent with running water	354	72%	0.45
	Percent with functional toilet	354	86%	0.35
Amenities:	Percent with air conditioning/fan	359	60%	0.49
	Percent of buildings requiring no rehabilitation	362	46%	0.50
**Process**				
Clinical competence:	Percent correct of 23 questions testing general clinical knowledge	362	57%	0.25
Referral process:	Communication level with referral facility (0 = Never, 1 = Seldom, 2 = Sometimes, 3 = Most times, 4 = Always)	354	1.18	1.62
	Percent offering transportation to referral facility	362	18%	0.39
Antenatal care quality:	Percent weighed at admittance	2,140	62%	0.17
	Percent height measured at admittance	2,140	54%	0.20
	Percent blood pressure measured	2,140	62%	0.18
	Percent urine sample taken	2,140	56%	0.19
	Percent blood sample taken	2,140	58%	0.18
	Percent stomach palpated	2,140	60%	0.18
	Percent uterine height measured	2,140	53%	0.20
	Percent blood type asked	2,140	45%	0.20
	Percent given dietary advice	2,140	59%	0.18
	Percent counseled on newborn baby care	2,140	58%	0.19
	Percent counseled on breastfeeding	2,140	59%	0.19
	Percent given HIV test	2,140	56%	0.19
	Percent counseled on pregnancy complications	2,140	58%	0.19
	Percent given tetanus injection	2,140	62%	0.18
	Percent given anti-malarial drugs	2,140	65%	0.16
	Percent given iron supplements	2,140	60%	0.16
Obstetric care quality:	Number of BeMONC services	354	4.14	1.68
	Percent offering caesarean section	355	5%	0.23
	Percent offering PMTCT	362	37%	0.48
Postnatal care quality:	Percent received post-natal reviews in 48-hrs	2,140	20%	0.40
**Outcomes**				
Neonatal:	Rate of neonatal deaths (per 1000 deliveries)	362	6.91	29.10
Obstetric:	Rate of obstetric complications (per delivery)	362	16.79	236.50
Maternal:	Rate of maternal deaths (per 1000 deliveries)	362	0.04	0.11

Notes: The indicators in the table were used to create the structure, process, and outcomes indices of facility quality, and the overall index of facility quality. Data come from the health facility survey (*N* = 362 except where otherwise noted) except for the measures of antenatal and postnatal quality which come from the women’s survey (*N* = 2140). The rates of neonatal deaths, obstetric complications, and maternal deaths were derived from facility records based on all deliveries at the facility in the 12 months preceding the survey. Clinical competence was measured using a multiple-choice test. Domains tested included antenatal care, labor and childbirth care, newborn care, and postpartum care. The test was administered to a randomly selected health worker in each health facility. BeMONC refers to Basic Emergency Obstetric and Newborn Care. PMTCT refers to Prevention of Mother to Child Transmission of HIV.

#### Does facility quality influence utilization of obstetric care?

Table 3 relates overall quality of care to utilization of antenatal and delivery care. In the first column, we examine how overall quality in a primary health facility relates to utilization of antenatal and delivery care in that facility. If higher quality is associated with greater utilization, a secondary question of interest is whether this might be because women are substituting from other health facilities or whether some of this increase is driven by new users. A priori, it is unclear whether higher quality at the primary health facility will lead women who would otherwise have traveled further away to use services to switch to the primary health care facility (particularly because care at private facilities and hospitals is more expensive), or whether higher quality in the primary health facility may draw in women who would not have used care at all (i.e., users of informal care). In the remaining columns in Table 3 we examine how quality at the primary health facility relates to utilization of obstetric care at other health facilities. Specifically, we examine how overall quality at the primary health facility relates to obstetric care utilization at other public facilities including general hospitals, at private facilities, and at any formal health facility. Obtaining a significant coefficient in the latter regression would indicate that higher quality in the primary health facility is associated with an overall increase in utilization.

**Table 3:** Care quality and the utilization of obstetric care.

	Primary Health Facility	Other Public Facility	Private Facility	Any Formal Facility
***Antenatal Care*:**				
Overall quality: general index (+ = better)	0.041***	-0.015*	-0.013**	0.006
	(0.014)	(0.009)	(0.007)	(0.006)
Number of observations	2,140	2,140	2,140	2,140
R-squared	0.228	0.132	0.083	0.059
F statistic	27.819	10.140	6.009	3.848
***Delivery Care*:**	0.029**	0.002	-0.014*	0.016
Overall quality: general index (+ = better)	(0.011)	(0.008)	(0.007)	(0.011)
Number of observations	2,140	2,140	2,140	2,140
R-squared	0.374	0.140	0.109	0.515
F statistic	90.045	14.002	7.746	307.178

Notes: The table describes the relationship between overall facility quality and utilization of antenatal and delivery care. Models are estimated using OLS. The dependent variables in each regression are reported in the table header (utilization of the primary health facility, utilization of other public facility, utilization of a private facility, utilization of any formal facility). The independent variables are normalized indices derived using principal component analysis applied to the structure, process, and outcomes index components. Each model includes as covariates potentially confounding individual and household characteristics. Robust standard errors clustered at the facility level are reported.

Significance:

*** p<0.01,

** p<0.05,

* p<0.1.

**Fig 1:**
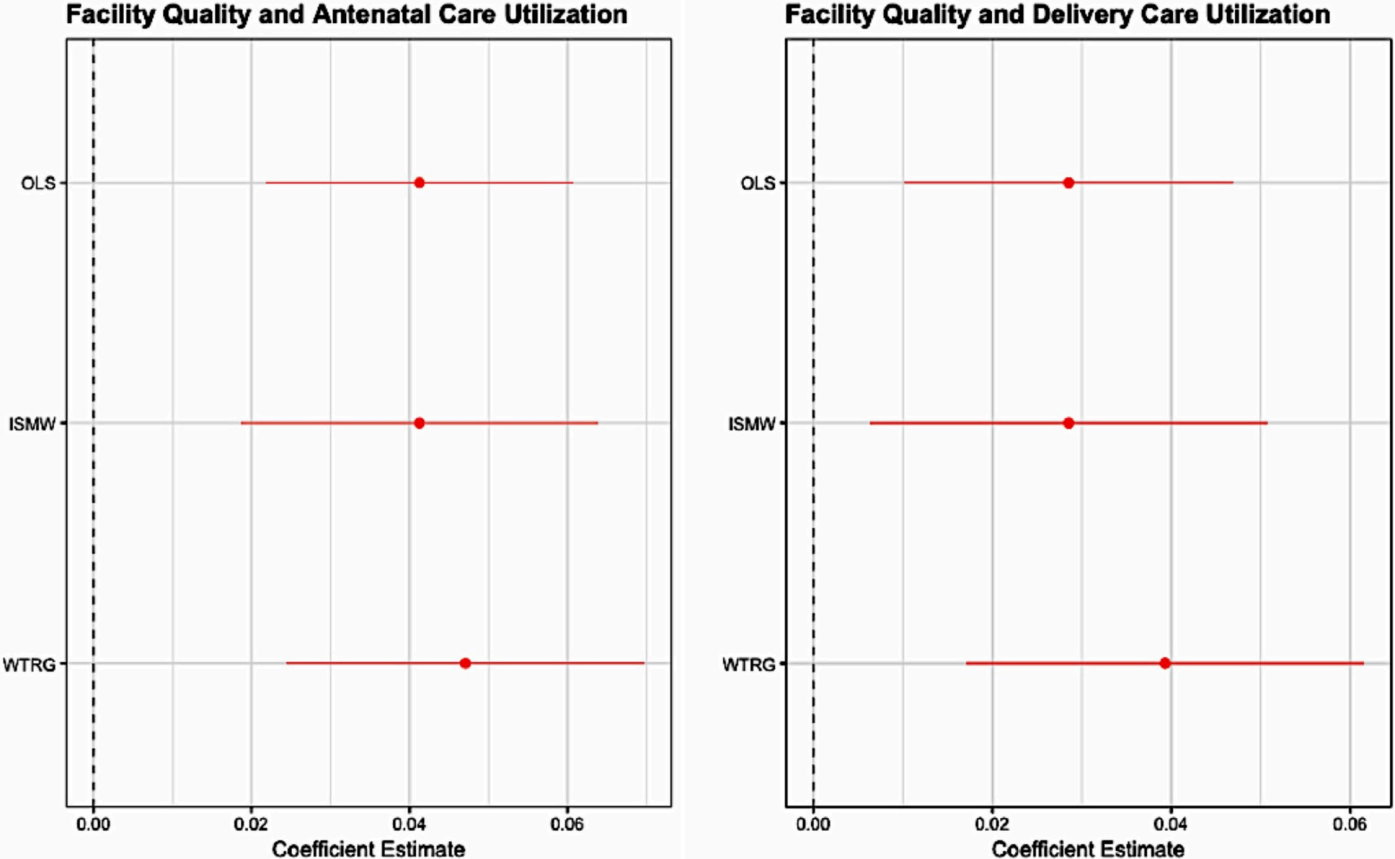
Facility quality and obstetric care utilization. The figure shows the OLS, ISMW, and WTRG estimates and 95% confidence intervals of the relationship between facility quality and antenatal and delivery care utilization based off n = 2,140 deliveries that occurred within 1 year of the facility survey.

We observe that higher overall quality is associated with significantly greater utilization of antenatal and delivery care at the primary health facility. A 1 standard deviation increase on the overall quality index is associated with a 4.1% and 2.9% increase in utilization of antenatal and delivery care, respectively at the primary health facility. There is some evidence of potential substitution behavior: higher quality at the primary health facility is associated with decreased utilization of care in other facilities (where care is more expensive). There is, however, no evidence of an increase in overall utilization at any formal facility. The propensity score estimates described in the Appendix (S1 File and Fig 1A and 1B) coincide to support the overall conclusion that higher facility quality leads to increased utilization of care, and provide evidence that the estimates are robust to model specification.

In Table 4, we examine whether the effect of quality on utilization of delivery care is stronger for women who attended antenatal care in the study facility. Women who attended antenatal care in the facility might gain first-hand information about facility quality and use that information when deciding where to obtain delivery care. We may therefore see a larger effect for such women. We indeed find evidence consistent with this. For women who received antenatal care at the primary health facility, a 1 standard deviation increase in overall quality of antenatal care is associated with 4.8% greater utilization of delivery care. This is driven by the process of care index. For women who received antenatal care at the primary health facility, a 1 standard deviation increase on the process index is associated with 7.5% greater utilization of delivery care.

**Table 4:** Care quality and delivery care after receiving antenatal care at the study facility.

	Delivery Care at the Primary Health Facility	
	Eq (1)	Eq (2)
***Overall:***		
Overall quality: general index (+ = better)	-0.023*	
	(0.013)	
Overall quality * Antenatal care at study facility	0.048***	
	(0.017)	
***Dimensions:***		
Structure: general index (+ = better)		0.020
		(0.012)
Process: general index (+ = better)		-0.046***
		(0.012)
Outcomes: general index (+ = better)		-0.001
		(0.016)
Structure index * Antenatal care at study facility		-0.019
		(0.017)
Process index * Antenatal care at study facility		0.075***
		(0.016)
Outcomes index * Antenatal care at study facility		0.025
		(0.022)
Number of observations	2,140	2,140
R-squared	0.598	0.603
F statistic	156.272	140.859

Notes: The table estimates the relationship between the quality indices and utilization of delivery care at the study facility for women who attended antenatal care at the same facility. Models are estimated using OLS. The dependent variable is a binary indicator denoting utilization of delivery care at the primary health facility. The independent variables are normalized indices derived using principal component analysis applied to the structure, process, and outcomes index components. Each model includes as covariates potentially confounding individual and household characteristics. Robust standard errors clustered at the facility level are reported.

Significance:

*** p<0.01,

** p<0.05,

* p<0.1

#### What dimensions of quality matter for utilization?

**Fig 2:**
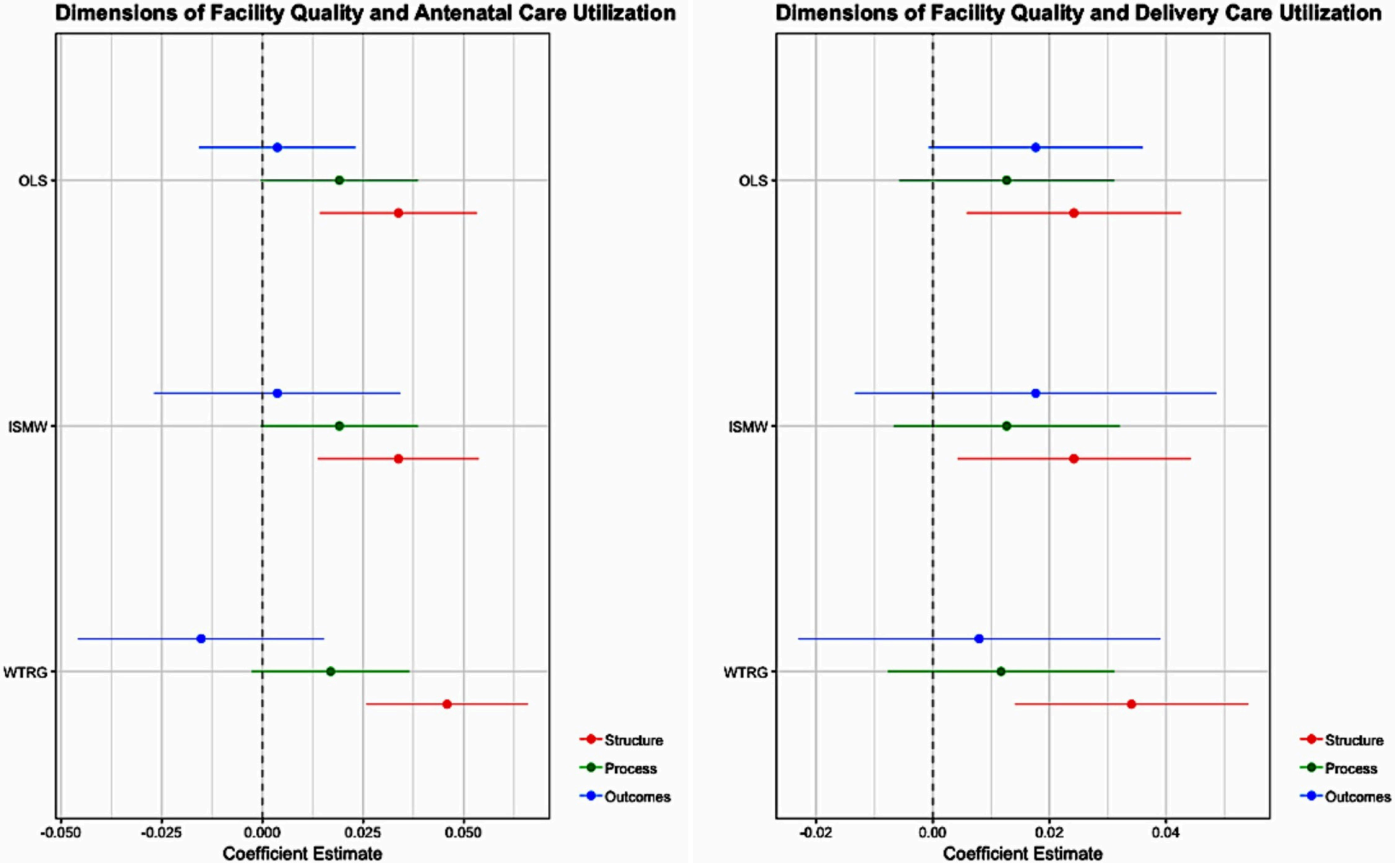
Dimensions of facility quality and obstetric care utilization. The figure shows the OLS, ISMW, and WTRG estimates and 95% confidence intervals of the relationship between structure, process, and outcomes measures of facility quality and antenatal and delivery care utilization based off n = 2,140 deliveries that occurred within 1 year of the facility survey.

In Table 5 we examine how each domain of quality relates to care utilization. For both antenatal and delivery care, structure is the dimension of quality that exhibits the largest and most statistically significant relationship with utilization. The estimates indicate that a 1 standard deviation increase on the structural quality index is associated with a 3.4% and 2.4% increase, respectively in utilization of antenatal and delivery care. In contrast, there is no observed relationship between the process and outcomes indices and care utilization. The coefficients in both cases are small and not statistically significant. The propensity score estimates described in the Appendix (and Fig 2A and 2B) confirm that the relationship between quality and utilization is driven by structural quality, and provide evidence that the estimates are robust to model specification.

**Table 5:** Dimensions of care quality and the utilization of obstetric care.

	Primary Health Facility
***Antenatal Care*:**	
Structure: general index (+ = better)	0.034**
	(0.014)
Process: general index (+ = better)	0.019
	(0.014)
Outcomes: general index (+ = better)	0.004
	(0.013)
Number of observations	2,140
R-squared	0.228
F statistic	25.167
***Delivery Care*:**	
Structure: general index (+ = better)	0.024**
	(0.011)
Process: general index (+ = better)	0.013
	(0.012)
Outcomes: general index (+ = better)	0.018
	(0.012)
Number of observations	2,140
R-squared	0.374
F statistic	83.294

Notes: The table shows the relationship between specific dimensions of facility quality and utilization of antenatal and delivery care in the study facility. Models are estimated using OLS. The independent variables are normalized indices derived using principal component analysis applied to the structure, process, and outcomes index components. Each model includes as covariates potentially confounding individual and household characteristics. Robust standard errors clustered at the facility level are reported.

Significance:

*** p<0.01,

** p<0.05,

* p<0.1

#### Is objective quality related to subjective perceptions of quality?

**Fig 3:**
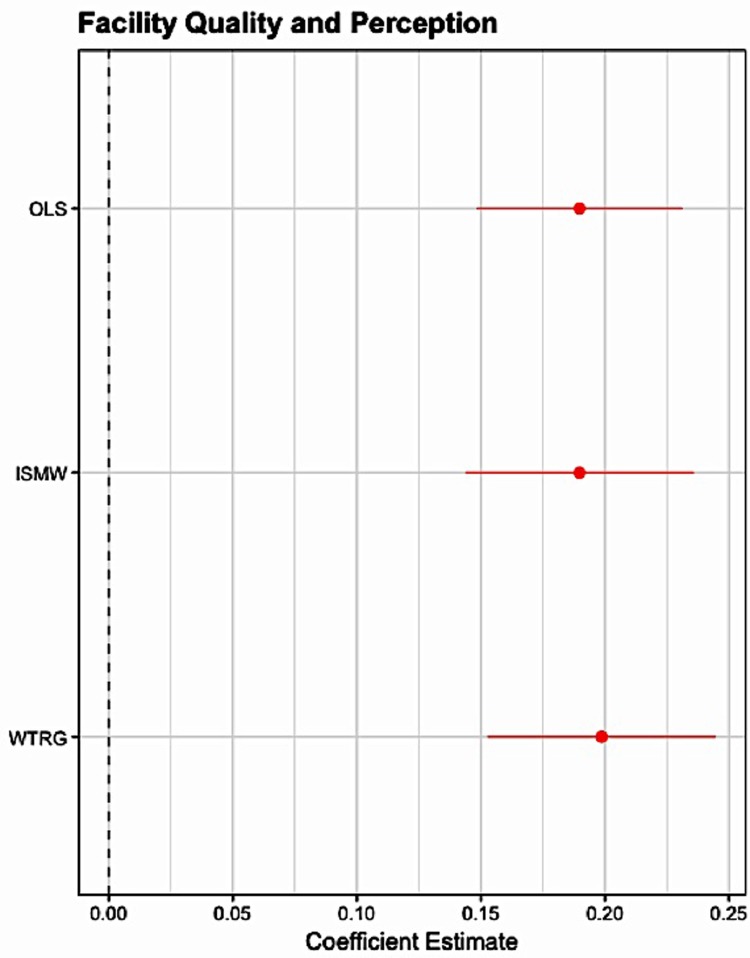
Objective facility quality and perceived facility quality. The figure shows the OLS, ISMW, and WTRG estimates and 95% confidence intervals of the relationship between facility quality and women’s perception of quality. The sample includes n = 2,140 deliveries that occurred within 1 year of the facility survey.

**Fig 4:**
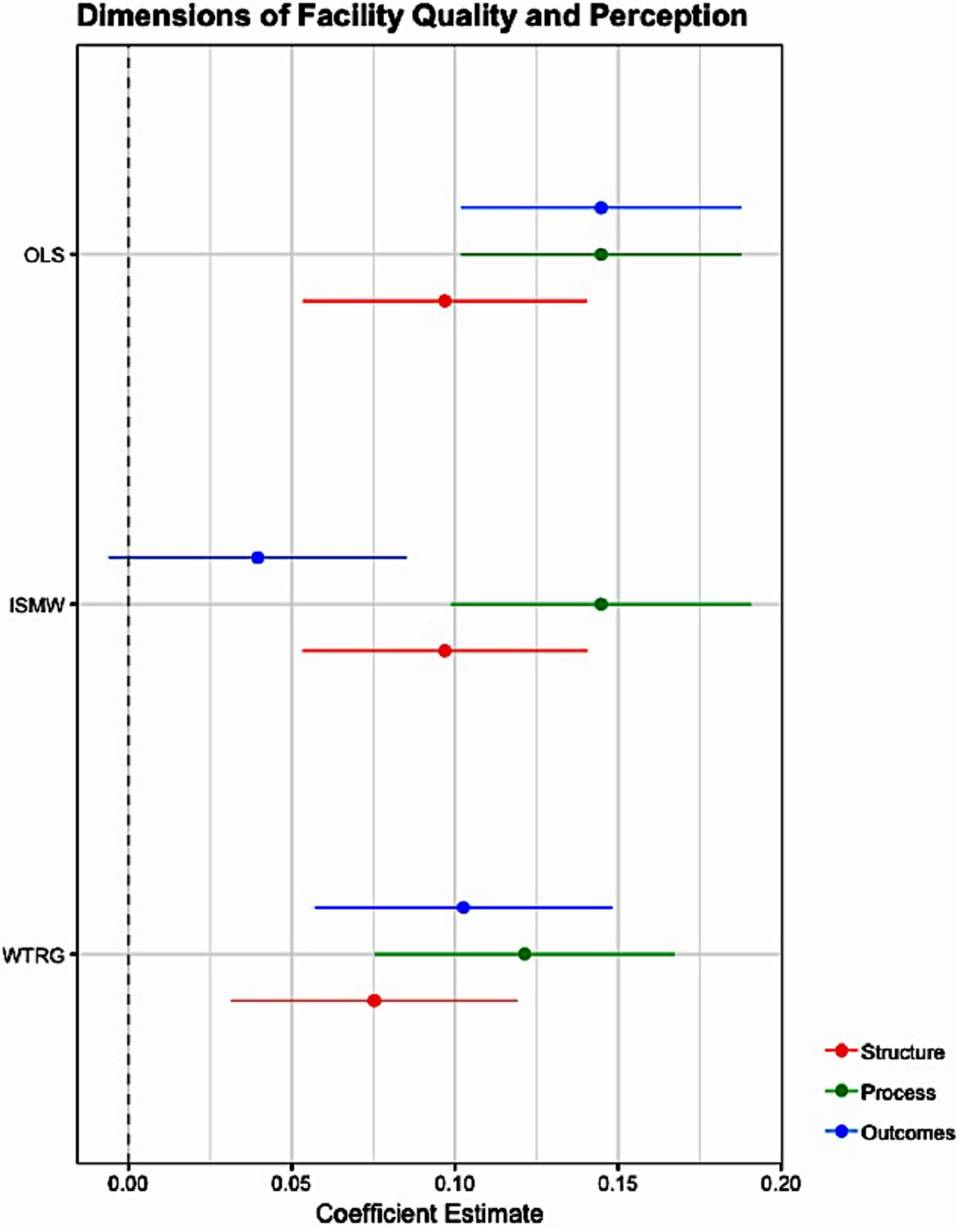
Dimensions of objective facility quality and perceived facility quality. The figure shows the OLS, ISMW, and WTRG estimates and 95% confidence intervals of the relationship between structure, process, and outcomes measures of facility quality and women’s perception of quality. The sample includes n = 2,140 deliveries that occurred within 1 year of the facility survey.

Table 6 relates overall quality to women’s perceptions of the quality of the primary health facility. For the overall quality index, the estimate is positive and significant, indicating that better objective facility quality is associated with higher subjective perceptions of facility quality. The coefficient indicates that a 1 standard deviation increase in facility quality is associated with a 0.19 standard deviation increase in women’s reported perception of the quality of care provided at the primary health facility. Table 6 also reports how the three dimensions of quality–structure, process, and outcomes–relate to women’s perception of the quality of care provided at the facility. While all three dimensions are positively related to perceived quality, only structure and process are statistically significant. The coefficients indicate that a 1 standard deviation increase in the structural dimension of quality is associated with a 0.097 standard deviation increase in perceived quality, and a 1 standard deviation increase in the process dimension of quality is associated with a 0.145 standard deviation increase in the perceived quality. The propensity score estimates described in the Appendix (and Figs 3 and 4.) confirm that perceived quality is significantly related to structural and process quality, and provide evidence that the estimates are robust to model specification.

**Table 6:** Quality of care and the perception of quality.

	Perception of Facility Quality	
	Eq (1)	Eq (2)
***Overall*:**		
Overall quality: general index (+ = better)	0.190***	
	(0.041)	
***Dimensions*:**		
Structure: general index (+ = better)		0.097***
		(0.036)
Process: general index (+ = better)		0.145***
		(0.038)
Outcomes: general index (+ = better)		0.040
		(0.029)
Number of observations	2,140	2,140
R-squared	0.074	0.076
F statistic	5.227	4.885

Notes: The table shows the relationship between objectively measured quality and women’s subjective perceptions of facility quality. Models are estimated using OLS. Perceptions are measured on four-point scale from 1: Poor to 4: Excellent. The independent variables are normalized indices derived using principal component analysis applied to the structure, process, and outcomes index components. Each model includes as covariates potentially confounding individual and household characteristics. Robust standard errors clustered at the facility level are reported.

Significance:

*** p<0.01,

** p<0.05,

* p<0.1

## Discussion

This paper addresses three questions: first, does quality of care influence utilization of obstetric care; second, if so, what dimensions of quality matter; and third, is objective quality related to subjective perceptions? Our results indicate that quality is positively correlated with utilization. Disaggregating by domains of quality suggests that the structural dimension of quality is the key driver of utilization. We also find that subjective perceptions are strongly related to objectively measured quality. We observe statistically significant relationships for structure and process but not for outcomes. The results are consistent across multiple empirical methods. Compared to the estimates generated using ordinary least squares described above, the propensity score estimates described in the Appendix only differ slightly in magnitude. The lack of model dependence lends credibility to the estimates, if not a causal interpretation. A causal interpretation of these estimates relies on the weak unconfoundedness assumption; specifically that assignment to treatment is as good as random within subpopulations that are homogenous in observed pre-treatment variables [38]. If this assumption holds, the estimates can be interpreted as causal.

The majority of studies that examine factors related to the utilization of obstetric care focus on sociocultural factors, perceived benefit/need of skilled attendance, economic accessibility, and physical accessibility [23, 39]. Few quantitative studies assess the relationship between quality of care and utilization. Both [25–26] found no significant association between structural quality indicators like the number of health workers on formal delivery care, while [27] found no relationship with availability of obstetric equipment. Our results align more closely with that of [22] and [28] who found a correlation between number of doctors and utilization. This study improves on earlier work by collecting considerably more detailed data on facility quality allowing us to not only better measure quality, but also to separate it into its various components to provide policy makers with evidence on which particular dimensions drive utilization.

A potential narrative for the results in this paper is that decisions regarding utilization of antenatal and delivery care are made based on what women can observe with greater certainty. Because the expected outcomes from utilizing antenatal and delivery care are uncertain prior to delivery, structural elements of the facility that can be easily observed (e.g. number of providers, general infrastructure) are more closely related to utilization. This narrative is supported by the fact that for women who used antenatal care at the facility, the process index exerts greater influence on their subsequent decisions to utilize delivery care. We may not observe a significant relationship between facility outcomes and utilization because these are much more difficult to observe and may also be more difficult to interpret. Outcomes are also a function of underlying risk characteristics.

Overall, the results generate some useful policy implications. A key implication is that if policy makers wish to improve utilization, quality investments should be made in areas that can be observed by households. This, of course, will need to be carefully weighed against investments that are more closely tied to outcomes. With regards to increasing utilization, it is noteworthy that in this study we do not find that higher quality in the primary health facility leads to an increase in overall levels of formal care utilization, but instead appears to lead to substitution from other health facilities (though given the cross-sectional nature of the data this is only suggestive not conclusive). This suggests that interventions targeting the demand-side will still be needed if the goal is to increase overall utilization. We emphasize, however, that while policy makers might be more exercised by overall levels, substitution may also be beneficial to the extent that households spend less on care allowing these savings to be reallocated to other goods. Substitution is also beneficial if households are substituting higher quality facilities for lower quality ones.

This study has some limitations. The first limitation is that the data on quality are cross-sectional. One concern is reverse causation. This could be the case if facilities with greater utilization receive more resources that can then be invested in structural quality. However, we do observe that, for plausibly informed women, process quality also influences utilization suggesting that causality runs from quality to utilization. The second limitation is that our measures of facility quality may not perfectly capture true quality. We use indices derived from multiple observed characteristics of the facility to measure quality. If the variables used to construct these indices are not accurate, the estimates may be biased. For instance, the estimated association between outcome quality and utilization could be biased toward zero if the clinical records of neonatal deaths, obstetric complications, and maternal deaths are inaccurate. The third limitation is that our data are observational and we do not have exogenous variation in facility quality. As a result, the estimates could be biased by unobserved heterogeneity. While we use multiple empirical methods that demonstrate similar relationships between quality and care utilization, the causal interpretation of each estimate relies on the same assumption of weak unconfoundedness. If the quality of the facilities varies systematically with unobserved characteristics of the sample, the point estimates will reflect these correlations.

## Conclusion

Proper obstetric care is critical for birth outcomes, yet remains underutilized in many low and middle-income countries. Using matching facility and household survey data from Nigeria, this paper has examined the association between health facility quality and utilization of obstetric care. We also explored whether subjective perceptions of quality carry information about objective quality. We find that quality of care is an important determinant of obstetric care utilization, though this relationship appears to be explained by more observable, structural dimensions of quality. We also find that perceptions of quality are strongly related to actual quality suggesting that perceptions carry important information. The results in this paper provide useful evidence to policy makers on the relationship between facility quality and demand for obstetric care.

## Supporting information

S1 File Appendix. (DOCX)

S2 File Better Obstetrics in Rural Nigeria (BORN) health facility survey. (PDF)

S3 File Better Obstetrics in Rural Nigeria (BORN) household survey. (PDF)
