# Supplementary material for: Utilization and quality: How the quality of care influences demand for obstetric care in Nigeria
Source: PLoS One. 2019 Feb 7;14(2):e0211500. doi: 10.1371/journal.pone.0211500 (PMC6366755; doi:10.1371/journal.pone.0211500)
Supplement: S1 File — (DOCX) [file pone.0211500.s001.docx]

**Appendix**

**Propensity Score Estimates**

As described in the main text, we supplement our main estimates with propensity score estimates. Propensity score methods have long been used for causal inference in observational studies to compare similar women exposed to different levels of quality. A key challenge in using propensity scores in this context is that we are interested in the effects of a continuous, rather than binary, treatment. Continuous treatments adopt slightly altered underlying assumptions to obtain a generalized propensity score [31]. Specifically, they assume that selection into levels of the treatment is random conditional on a rich set of observable covariates, or generalized propensity score. This assumption enables the estimation of an average dose-response-function (ADRF) relating a continuously valued level of treatment exposure to the outcome [32].

The first step in this procedure is to estimate the conditional expectation of the outcome, $U_{i}$, as a function of the observed treatment level, $t_{i}$, and the generalized propensity score $r_{i}$. The second step is to estimate a value of the dose-response function by averaging $\mu(t_{i}, r_{i})$, the conditional mean of the outcome at treatment level $t_{i}$ and generalized propensity score $r_{i}$, at that particular levels of the treatment. This yields the ADRF, or $\beta(t_{i})$. Research demonstrates that, under the weak unconfoundedness assumption, estimating the ADRF by adjusting for the generalized propensity score in this way removes the selection bias [31]. Ultimately, the weak unconfoundedness assumption implies that assignment to the level of treatment is unconfounded conditional on the generalized propensity score. This assumption prohibits systematic selection into levels of the treatment based on unobservable characteristics.

First, we examine whether facility quality influences obstetric care utilization. Fig. A.1.a. and Fig. A.1.b. compare the OLS estimates described in Table 3 to propensity score estimates using both ISMW and WTRG methods. For both antenatal and delivery care utilization, the ISMW estimate is essentially equal to the OLS estimate, while the WTRG estimate is slightly larger (4.7% vs. 4.1% in the case of antenatal care, and 3.9% vs. 2.9% in the case of delivery care). In general, the differences in magnitude are minor, and the direction and significance are consistent. These estimates support the overall conclusion that higher facility quality leads to increased utilization of care, and provide evidence that the estimates are robust to model specification.

**Fig A.1.a. and Fig A.1.b.: Facility Quality and Obstetric Care Utilization** The figure shows the OLS, ISMW, and WTRG estimates and 95% confidence intervals of the relationship between facility quality and antenatal and delivery care utilization based off n=2,140 deliveries that occurred within 1 year of the facility survey.

Next, we examine how each domain of quality relates to care utilization. Fig. A.2.a. and Fig. A.2.b. compare the OLS estimates described in Table 5 to the ISMW and WTRG estimates. For the structural index, the ISMW estimate is essentially equal to the OLS estimate for both outcomes, while the WTRG estimate is slightly larger (4.5% vs. 3.3% in the case of antenatal care, and 3.4% vs. 2.4% in the case of delivery care). For the process index, both the ISMW and WTRG estimates are essentially equal to the OLS estimate for both outcomes (1.9% for OLS and ISMW vs. 1.7% for WTRG in the case of antenatal care, and 1.3% for OLS and ISMW vs. 1.2% for WTRG in the case of delivery care). For the outcomes index, the ISMW estimate is essentially equal to the OLS estimate for both outcomes, while the WTRG estimate is smaller (0.3% vs. -1.5% in the case of antenatal care, and 1.7% vs. 0.8% in the case of facility deliveries). In general, the differences in magnitude are minor while the direction and significance are consistent in most cases except for the relationship between the outcomes index and antenatal care. These estimates support the overall conclusion that the relationship between quality and utilization is driven by structural quality, and provide evidence that the estimates are robust to model specification.

**Fig A.2.a. and Fig A.2.b.: Dimensions of Facility Quality and Obstetric Care Utilization** The figure shows the OLS, ISMW, and WTRG estimates and 95% confidence intervals of the relationship between structure, process, and outcomes measures of facility quality and antenatal and delivery care utilization based off n=2,140 deliveries that occurred within 1 year of the facility survey.

Next we examine how overall quality relates to women’s perceptions of quality. Figure A.3. compares the OLS estimate described in Table 6 to the ISMW and WTRG propensity score estimates. As shown, the estimated relationship between overall quality and women’s perception of the quality of care provided are essentially the same across the different methods. These estimates support the conclusion is that overall quality is positively related to perceptions of quality, and provide evidence that the estimates are robust to model specification.

**Fig A.3.: Objective Facility Quality and Perceived Facility Quality** The figure shows the OLS, ISMW, and WTRG estimates and 95% confidence intervals of the relationship between facility quality and women’s perception of quality. The sample includes n=2,140 deliveries that occurred within 1 year of the facility survey.

Finally, we examine how the three dimensions of quality – structure, process, and outcomes – relate to women’s perception of quality. Figure A.4. compares the OLS estimates for the three dimensions of quality described in Table 6 to the ISMW and WTRG propensity score estimates. The estimated relationship between structural quality and women’s perception of the quality of care provided are essentially the same for the OLS and ISMW methods (0.097), while the WTRG estimate is slightly, though not significantly, different (0.075). Similarly, the OLS and ISMW estimates of the relationship between process quality and women’s perception are equal (0.145), while the WTRG estimate is different (0.12) but not significantly so. For the association between outcome quality and perceptions, the OLS estimate is 0.145, the ISMW estimate is 0.040, and the WRTG estimate is 0.103. These estimates support the overall conclusion that perceived quality is significantly related to structural and process quality, and provide evidence that the estimates are robust to model specification.

**Fig A.4.: Dimensions of Objective Facility Quality and Perceived Facility Quality** The figure shows the OLS, ISMW, and WTRG estimates and 95% confidence intervals of the relationship between structure, process, and outcomes measures of facility quality and women’s perception of quality. The sample includes n=2,140 deliveries that occurred within 1 year of the facility survey.

**Summary Statistics of the Overall, Structure, Process, and Outcome Quality Measures**

The following tables describe the correlations between the overall index of quality, the structure, process, and outcome indices of quality, and the variables used in generating the indices.

**Table A.1.a.: Correlation Matrix of the Overall Index, Structure Index, Sub-indices and Basic Measures**

|  | **Overall Index** | **Index** | **Sub-indices** | | | | | |
| --- | --- | --- | --- | --- | --- | --- | --- | --- |
| **Overall Index** | Care quality | Structure | Size | Provid- ers | Equipment | Clinical inf. | General inf. | Amenit-ies |
| Overall care quality | 1.00 |  |  |  |  |  |  |  |
|  |  |  |  |  |  |  |  |  |
| **Index** |  |  |  |  |  |  |  |  |
| Structure | 0.77 | 1.00 |  |  |  |  |  |  |
|  |  |  |  |  |  |  |  |  |
| **Sub-indices** |  |  |  |  |  |  |  |  |
| Size | 0.47 | 0.59 | 1.00 |  |  |  |  |  |
| Providers | 0.48 | 0.44 | 0.26 | 1.00 |  |  |  |  |
| Equipment | 0.44 | 0.58 | 0.19 | 0.16 | 1.00 |  |  |  |
| Clinical infrastructure | 0.40 | 0.57 | 0.36 | -0.02 | 0.22 | 1.00 |  |  |
| General infrastructure | 0.41 | 0.62 | 0.13 | 0.07 | 0.19 | 0.22 | 1.00 |  |
| Amenities | 0.39 | 0.57 | 0.06 | 0.02 | 0.21 | 0.16 | 0.46 | 1.00 |
|  |  |  |  |  |  |  |  |  |
| **Basic Measures** |  |  |  |  |  |  |  |  |
| Number of beds | 0.29 | 0.44 | 0.72 | 0.02 | 0.22 | 0.25 | 0.15 | 0.14 |
| Number of staff | 0.39 | 0.41 | 0.73 | 0.35 | 0.06 | 0.27 | 0.03 | -0.05 |
| Number of doctors | 0.17 | 0.12 | 0.29 | 0.28 | -0.01 | -0.06 | -0.06 | -0.04 |
| Number of nurses | 0.17 | 0.19 | 0.26 | 0.28 | 0.08 | -0.08 | 0.02 | 0.08 |
| Number of midwives | 0.27 | 0.20 | 0.24 | 0.40 | 0.09 | 0.02 | 0.00 | -0.06 |
| Percent with 24hr/7day provider availability | 0.35 | 0.35 | 0.13 | 0.83 | 0.12 | -0.03 | 0.09 | 0.04 |
| Percent with 24hr/7day delivery services | 0.36 | 0.34 | 0.09 | 0.80 | 0.14 | 0.02 | 0.07 | 0.01 |
| Percent with supply of medicines | 0.07 | 0.16 | 0.07 | -0.03 | 0.22 | 0.30 | 0.03 | -0.06 |
| Percent with adult weight scale | 0.24 | 0.34 | 0.14 | 0.14 | 0.59 | 0.10 | 0.10 | 0.09 |
| Percent with baby weight scale | 0.26 | 0.38 | 0.12 | 0.15 | 0.53 | 0.11 | 0.21 | 0.15 |
| Percent with delivery bed | 0.31 | 0.38 | 0.17 | 0.08 | 0.58 | 0.17 | 0.11 | 0.17 |
| Percent with midwifery kit | 0.14 | 0.09 | -0.01 | 0.09 | 0.37 | -0.05 | -0.08 | 0.00 |
| Percent with delivery kit | 0.22 | 0.28 | 0.04 | 0.02 | 0.52 | 0.08 | 0.15 | 0.14 |
| Percent with incubator | 0.10 | 0.16 | 0.04 | -0.03 | 0.31 | 0.07 | 0.04 | 0.11 |
| Percent with laboratory | 0.33 | 0.47 | 0.36 | -0.01 | 0.15 | 0.80 | 0.18 | 0.12 |
| Percent with pharmacy | 0.19 | 0.37 | 0.18 | -0.05 | 0.20 | 0.72 | 0.13 | 0.06 |
| Percent with functional ambulance | 0.30 | 0.29 | 0.17 | 0.04 | 0.05 | 0.44 | 0.13 | 0.16 |
| Percent with electricity grid connection | 0.29 | 0.32 | 0.04 | 0.16 | 0.11 | 0.16 | 0.33 | 0.26 |
| Percent with functional generator | 0.23 | 0.39 | 0.14 | 0.01 | 0.10 | 0.18 | 0.64 | 0.24 |
| Percent with running water | 0.15 | 0.29 | 0.02 | -0.01 | 0.03 | 0.06 | 0.60 | 0.28 |
| Percent with functional toilet | 0.33 | 0.48 | 0.09 | 0.07 | 0.22 | 0.15 | 0.75 | 0.33 |
| Percent with air conditioning/fan | 0.33 | 0.49 | 0.04 | 0.02 | 0.23 | 0.14 | 0.44 | 0.79 |
| Percent of buildings requiring no rehabilitation | 0.30 | 0.40 | 0.05 | 0.01 | 0.11 | 0.11 | 0.29 | 0.79 |
|  |  |  |  |  |  |  |  |  |

**Table A.1.b.: Correlation Matrix of the Overall Index, Structure Index, Sub-indices and Basic Measures**

|  | **Basic Measures** | | | | | | | |
| --- | --- | --- | --- | --- | --- | --- | --- | --- |
| **Basic Measures** | Beds | Staff | Doctors | Nurses | Midwi-ves | 24/7 provider | 24/7 delivery | Medic-ines |
| Number of beds | 1.00 |  |  |  |  |  |  |  |
| Number of staff | 0.05 | 1.00 |  |  |  |  |  |  |
| Number of doctors | 0.03 | 0.39 | 1.00 |  |  |  |  |  |
| Number of nurses | 0.02 | 0.35 | 0.17 | 1.00 |  |  |  |  |
| Number of midwives | -0.12 | 0.47 | 0.10 | 0.13 | 1.00 |  |  |  |
| Percent with 24hr/7day provider availability | 0.08 | 0.10 | 0.07 | 0.02 | 0.21 | 1.00 |  |  |
| Percent with 24hr/7day delivery services | -0.01 | 0.14 | 0.05 | 0.08 | 0.06 | 0.54 | 1.00 |  |
| Percent with supply of medicines | 0.06 | 0.04 | 0.08 | -0.06 | 0.06 | -0.04 | -0.05 | 1.00 |
| Percent with adult weight scale | 0.13 | 0.07 | -0.05 | 0.04 | 0.04 | 0.12 | 0.14 | 0.06 |
| Percent with baby weight scale | 0.09 | 0.08 | -0.08 | 0.08 | 0.11 | 0.13 | 0.12 | 0.00 |
| Percent with delivery bed | 0.19 | 0.06 | 0.02 | 0.06 | -0.04 | 0.06 | 0.09 | 0.04 |
| Percent with midwifery kit | -0.04 | 0.02 | 0.01 | 0.10 | 0.19 | 0.01 | 0.04 | -0.01 |
| Percent with delivery kit | 0.13 | -0.07 | 0.06 | 0.02 | -0.02 | 0.03 | 0.00 | 0.08 |
| Percent with incubator | 0.10 | -0.04 | -0.02 | -0.06 | 0.00 | -0.04 | 0.02 | 0.03 |
| Percent with laboratory | 0.27 | 0.25 | -0.06 | -0.12 | -0.03 | -0.02 | 0.06 | 0.13 |
| Percent with pharmacy | 0.06 | 0.20 | -0.06 | 0.00 | 0.13 | -0.07 | -0.07 | 0.42 |
| Percent with functional ambulance | 0.19 | 0.05 | 0.02 | -0.05 | -0.09 | 0.05 | 0.08 | 0.02 |
| Percent with electricity grid connection | -0.13 | 0.20 | 0.02 | 0.03 | 0.15 | 0.09 | 0.15 | 0.05 |
| Percent with functional generator | 0.16 | 0.04 | -0.01 | 0.02 | 0.01 | 0.03 | -0.03 | 0.05 |
| Percent with running water | 0.11 | -0.08 | -0.13 | -0.03 | -0.07 | 0.05 | 0.01 | -0.04 |
| Percent with functional toilet | 0.12 | 0.01 | -0.01 | 0.03 | -0.03 | 0.07 | 0.08 | 0.03 |
| Percent with air conditioning/fan | 0.08 | -0.02 | 0.00 | 0.04 | 0.01 | 0.04 | -0.01 | 0.00 |
| Percent of buildings requiring no rehabilitation | 0.15 | -0.07 | -0.06 | 0.08 | -0.10 | 0.03 | 0.03 | -0.10 |
|  |  |  |  |  |  |  |  |  |

**Table A.1.c.: Correlation Matrix of the Overall Index, Structure Index, Sub-indices and Basic Measures**

|  | **Basic Measures** | | | | | | | |
| --- | --- | --- | --- | --- | --- | --- | --- | --- |
| **Basic Measures** | Adult scale | Baby scale | Delivery bed | Midwife kit | Delivery kit | Incuba-tor | Labora-tory | Pharma-cy |
| Percent with adult weight scale | 1.00 |  |  |  |  |  |  |  |
| Percent with baby weight scale | 0.23 | 1.00 |  |  |  |  |  |  |
| Percent with delivery bed | 0.12 | 0.17 | 1.00 |  |  |  |  |  |
| Percent with midwifery kit | 0.10 | 0.05 | 0.02 | 1.00 |  |  |  |  |
| Percent with delivery kit | 0.09 | 0.03 | 0.19 | 0.11 | 1.00 |  |  |  |
| Percent with incubator | 0.05 | 0.05 | 0.05 | 0.08 | 0.05 | 1.00 |  |  |
| Percent with laboratory | 0.08 | 0.04 | 0.12 | -0.02 | 0.13 | 0.02 | 1.00 |  |
| Percent with pharmacy | 0.07 | 0.13 | 0.14 | -0.01 | 0.02 | 0.02 | 0.29 | 1.00 |
| Percent with functional ambulance | 0.04 | 0.05 | 0.06 | -0.10 | -0.03 | 0.14 | 0.16 | 0.04 |
| Percent with electricity grid connection | 0.04 | 0.13 | 0.07 | 0.04 | 0.01 | -0.01 | 0.12 | 0.16 |
| Percent with functional generator | 0.07 | 0.10 | 0.01 | -0.11 | 0.16 | -0.01 | 0.16 | 0.11 |
| Percent with running water | 0.05 | 0.09 | -0.03 | -0.08 | 0.03 | 0.02 | 0.05 | 0.00 |
| Percent with functional toilet | 0.07 | 0.19 | 0.18 | -0.02 | 0.13 | 0.07 | 0.12 | 0.09 |
| Percent with air conditioning/fan | 0.09 | 0.11 | 0.17 | 0.04 | 0.18 | 0.07 | 0.07 | 0.12 |
| Percent of buildings requiring no rehabilitation | 0.05 | 0.13 | 0.10 | -0.03 | 0.03 | 0.10 | 0.12 | -0.03 |
|  |  |  |  |  |  |  |  |  |

**Table A.1.d.: Correlation Matrix of the Overall Index, Structure Index, Sub-indices and Basic Measures**

|  | **Basic Measures** | | | | | | |
| --- | --- | --- | --- | --- | --- | --- | --- |
| **Basic Measures** | Ambul-ance | Grid | Generator | Water | Toilet | AC/Fan | Buildings |
| Percent with functional ambulance | 1.00 |  |  |  |  |  |  |
| Percent with electricity grid connection | 0.00 | 1.00 |  |  |  |  |  |
| Percent with functional generator | 0.08 | 0.03 | 1.00 |  |  |  |  |
| Percent with running water | 0.11 | 0.07 | 0.13 | 1.00 |  |  |  |
| Percent with functional toilet | 0.09 | 0.10 | 0.26 | 0.22 | 1.00 |  |  |
| Percent with air conditioning/fan | 0.11 | 0.31 | 0.25 | 0.23 | 0.31 | 1.00 |  |
| Percent of buildings requiring no rehabilitation | 0.16 | 0.10 | 0.14 | 0.21 | 0.22 | 0.26 | 1.00 |
|  |  |  |  |  |  |  |  |
| Notes: The number of observations of each index and basic measure is 362, the number of facilities surveyed. This table is meant to show the correlation between the indices generated through principal component analysis and their input variables. The overall index of care quality was created through principal component analysis using the structure, process, and outcomes indices. The structure index was the result of principal component analysis using each of the structural sub-indices, specifically size, providers, equipment, clinical infrastructure, general infrastructure, and amenities. Each of the structural sub-indices were generated through principal component analysis using the basic measures described in this table. | | | | | | | |

**Table A.2.a.: Correlation Matrix of the Overall Index, Process Index, Sub-indices and Basic Measures**

|  | **Overall Index** | **Index** | **Sub-indices** | | | | |
| --- | --- | --- | --- | --- | --- | --- | --- |
| **Overall Index** | Care quality | Process | Clinic comp. | Referrals | ANC quality | Obs. quality | PNC quality |
| Overall care quality | 1.00 |  |  |  |  |  |  |
|  |  |  |  |  |  |  |  |
| **Index** |  |  |  |  |  |  |  |
| Process | 0.79 | 1.00 |  |  |  |  |  |
|  |  |  |  |  |  |  |  |
| **Sub-indices** |  |  |  |  |  |  |  |
| Clinical competence | 0.40 | 0.49 | 1.00 |  |  |  |  |
| Referral process | 0.13 | 0.35 | -0.17 | 1.00 |  |  |  |
| Antenatal care quality | 0.13 | 0.11 | -0.11 | -0.03 | 1.00 |  |  |
| Obstetric care quality | 0.47 | 0.56 | 0.08 | 0.07 | -0.09 | 1.00 |  |
| Postnatal care quality | 0.44 | 0.43 | 0.02 | -0.20 | 0.18 | -0.10 | 1.00 |
|  |  |  |  |  |  |  |  |
| **Basic Measures** |  |  |  |  |  |  |  |
| Test score | 0.13 | 0.11 | -0.11 | -0.03 | 1.00 | -0.09 | 0.18 |
| Communication level with referral facility | 0.35 | 0.46 | 0.11 | 0.12 | -0.06 | 0.74 | -0.09 |
| Percent offering transportation to referral facility | 0.34 | 0.36 | 0.01 | -0.02 | -0.07 | 0.73 | -0.05 |
| Percent weighed at admittance | 0.37 | 0.46 | 0.92 | -0.16 | -0.06 | 0.09 | 0.00 |
| Percent height measured at admittance | 0.35 | 0.43 | 0.83 | -0.17 | -0.20 | 0.11 | 0.05 |
| Percent blood pressure measured | 0.40 | 0.49 | 0.96 | -0.17 | -0.09 | 0.09 | 0.05 |
| Percent urine sample taken | 0.38 | 0.44 | 0.93 | -0.21 | -0.09 | 0.02 | 0.08 |
| Percent blood sample taken | 0.37 | 0.42 | 0.93 | -0.23 | -0.11 | 0.03 | 0.07 |
| Percent stomach palpated | 0.36 | 0.42 | 0.90 | -0.20 | -0.01 | 0.03 | 0.03 |
| Percent uterine height measured | 0.36 | 0.40 | 0.89 | -0.19 | -0.10 | 0.02 | 0.04 |
| Percent blood type asked | 0.38 | 0.39 | 0.84 | -0.18 | -0.14 | 0.07 | 0.02 |
| Percent given dietary advice | 0.39 | 0.45 | 0.95 | -0.18 | -0.10 | 0.06 | 0.03 |
| Percent counseled on newborn baby care | 0.38 | 0.49 | 0.95 | -0.13 | -0.13 | 0.10 | 0.01 |
| Percent counseled on breastfeeding | 0.37 | 0.50 | 0.94 | -0.09 | -0.14 | 0.11 | -0.01 |
| Percent given HIV test | 0.36 | 0.44 | 0.90 | -0.12 | -0.13 | 0.08 | -0.01 |
| Percent counseled on pregnancy complications | 0.36 | 0.46 | 0.95 | -0.15 | -0.12 | 0.07 | 0.01 |
| Percent given tetanus injection | 0.33 | 0.46 | 0.87 | -0.09 | -0.05 | 0.10 | 0.00 |
| Percent given anti-malarial drugs | 0.35 | 0.45 | 0.88 | -0.16 | -0.04 | 0.08 | 0.02 |
| Percent given iron supplements | 0.33 | 0.45 | 0.87 | -0.07 | -0.10 | 0.11 | -0.06 |
| Number of BeMONC services | 0.36 | 0.28 | 0.01 | -0.20 | 0.04 | -0.05 | 0.74 |
| Percent offering caesarean section | 0.18 | 0.12 | 0.10 | -0.04 | -0.01 | 0.13 | 0.01 |
| Percent offering PMTCT | 0.30 | 0.35 | 0.03 | -0.10 | 0.23 | -0.09 | 0.75 |
| Percent received post-natal reviews in 48-hrs | 0.13 | 0.35 | -0.17 | 1.00 | -0.03 | 0.07 | -0.20 |
|  |  |  |  |  |  |  |  |

**Table A.2.b.: Correlation Matrix of the Overall Index, Process Index, Sub-indices and Basic Measures**

|  | **Basic Measures** | | | | | | | |
| --- | --- | --- | --- | --- | --- | --- | --- | --- |
| **Basic Measures** | Test score | Comm. ref. | Trans. ref | Weighed | Height | BP | Urine samp. | Blood samp. |
| Test score | 1.00 |  |  |  |  |  |  |  |
| Communication level with referral facility | -0.06 | 1.00 |  |  |  |  |  |  |
| Percent offering transportation to referral facility | -0.07 | 0.08 | 1.00 |  |  |  |  |  |
| Percent weighed at admittance | -0.06 | 0.12 | 0.01 | 1.00 |  |  |  |  |
| Percent height measured at admittance | -0.20 | 0.13 | 0.03 | 0.79 | 1.00 |  |  |  |
| Percent blood pressure measured | -0.09 | 0.10 | 0.02 | 0.93 | 0.83 | 1.00 |  |  |
| Percent urine sample taken | -0.09 | 0.04 | -0.01 | 0.83 | 0.83 | 0.88 | 1.00 |  |
| Percent blood sample taken | -0.11 | 0.03 | 0.00 | 0.83 | 0.78 | 0.89 | 0.92 | 1.00 |
| Percent stomach palpated | -0.01 | 0.06 | -0.02 | 0.81 | 0.67 | 0.84 | 0.81 | 0.87 |
| Percent uterine height measured | -0.10 | 0.05 | -0.02 | 0.75 | 0.76 | 0.80 | 0.88 | 0.86 |
| Percent blood type asked | -0.14 | 0.12 | -0.01 | 0.68 | 0.69 | 0.76 | 0.83 | 0.84 |
| Percent given dietary advice | -0.10 | 0.09 | 0.00 | 0.86 | 0.76 | 0.89 | 0.85 | 0.87 |
| Percent counseled on newborn baby care | -0.13 | 0.13 | 0.02 | 0.87 | 0.78 | 0.92 | 0.83 | 0.86 |
| Percent counseled on breastfeeding | -0.14 | 0.13 | 0.03 | 0.85 | 0.75 | 0.90 | 0.81 | 0.84 |
| Percent given HIV test | -0.13 | 0.08 | 0.05 | 0.77 | 0.69 | 0.85 | 0.81 | 0.83 |
| Percent counseled on pregnancy complications | -0.12 | 0.08 | 0.02 | 0.86 | 0.77 | 0.89 | 0.88 | 0.87 |
| Percent given tetanus injection | -0.05 | 0.15 | -0.01 | 0.78 | 0.65 | 0.83 | 0.76 | 0.76 |
| Percent given anti-malarial drugs | -0.04 | 0.13 | -0.01 | 0.85 | 0.70 | 0.83 | 0.80 | 0.79 |
| Percent given iron supplements | -0.10 | 0.13 | 0.03 | 0.83 | 0.65 | 0.80 | 0.78 | 0.76 |
| Number of BeMONC services | 0.04 | -0.07 | 0.00 | 0.01 | 0.04 | 0.03 | 0.05 | 0.04 |
| Percent offering caesarean section | -0.01 | 0.22 | -0.02 | 0.14 | 0.15 | 0.12 | 0.08 | 0.07 |
| Percent offering PMTCT | 0.23 | -0.05 | -0.08 | 0.00 | 0.05 | 0.05 | 0.08 | 0.07 |
| Percent received post-natal reviews in 48-hrs | -0.03 | 0.12 | -0.02 | -0.16 | -0.17 | -0.17 | -0.21 | -0.23 |
|  |  |  |  |  |  |  |  |  |

**Table A.2.c.: Correlation Matrix of the Overall Index, Process Index, Sub-indices and Basic Measures**

|  | **Basic Measures** | | | | | | | |
| --- | --- | --- | --- | --- | --- | --- | --- | --- |
| **Basic Measures** | Stomach | Ut. height | Blood type | Diet advice | Baby care | Breastfeed | HIV test | Complic. |
| Percent stomach palpated | 1.00 |  |  |  |  |  |  |  |
| Percent uterine height measured | 0.84 | 1.00 |  |  |  |  |  |  |
| Percent blood type asked | 0.74 | 0.84 | 1.00 |  |  |  |  |  |
| Percent given dietary advice | 0.87 | 0.84 | 0.77 | 1.00 |  |  |  |  |
| Percent counseled on newborn baby care | 0.84 | 0.80 | 0.77 | 0.94 | 1.00 |  |  |  |
| Percent counseled on breastfeeding | 0.82 | 0.76 | 0.75 | 0.92 | 0.97 | 1.00 |  |  |
| Percent given HIV test | 0.76 | 0.78 | 0.77 | 0.85 | 0.88 | 0.89 | 1.00 |  |
| Percent counseled on pregnancy complications | 0.84 | 0.84 | 0.78 | 0.92 | 0.92 | 0.91 | 0.89 | 1.00 |
| Percent given tetanus injection | 0.80 | 0.71 | 0.70 | 0.80 | 0.80 | 0.81 | 0.74 | 0.79 |
| Percent given anti-malarial drugs | 0.78 | 0.74 | 0.71 | 0.81 | 0.81 | 0.80 | 0.76 | 0.83 |
| Percent given iron supplements | 0.78 | 0.71 | 0.64 | 0.82 | 0.81 | 0.82 | 0.75 | 0.82 |
| Number of BeMONC services | 0.01 | 0.00 | 0.01 | 0.00 | -0.01 | -0.03 | 0.00 | 0.01 |
| Percent offering caesarean section | 0.08 | 0.08 | 0.11 | 0.11 | 0.10 | 0.07 | 0.06 | 0.08 |
| Percent offering PMTCT | 0.04 | 0.06 | 0.03 | 0.05 | 0.03 | 0.02 | -0.02 | 0.01 |
| Percent received post-natal reviews in 48-hrs | -0.20 | -0.19 | -0.18 | -0.18 | -0.13 | -0.09 | -0.12 | -0.15 |
|  |  |  |  |  |  |  |  |  |

**Table A.2.d.: Correlation Matrix of the Overall Index, Process Index, Sub-indices and Basic Measures**

|  | **Basic Measures** | | | | | | |
| --- | --- | --- | --- | --- | --- | --- | --- |
| **Basic Measures** | Tetanus | Anti-malarial | Iron supp. | BeMONC | C-section | PMTCT | Post-natal rev. |
| Percent given tetanus injection | 1.00 |  |  |  |  |  |  |
| Percent given anti-malarial drugs | 0.81 | 1.00 |  |  |  |  |  |
| Percent given iron supplements | 0.82 | 0.82 | 1.00 |  |  |  |  |
| Number of BeMONC services | -0.03 | 0.02 | -0.03 | 1.00 |  |  |  |
| Percent offering caesarean section | 0.08 | 0.12 | 0.08 | 0.07 | 1.00 |  |  |
| Percent offering PMTCT | 0.03 | 0.02 | -0.05 | 0.11 | -0.01 | 1.00 |  |
| Percent received post-natal reviews in 48-hrs | -0.09 | -0.16 | -0.07 | -0.20 | -0.04 | -0.10 | 1.00 |
|  |  |  |  |  |  |  |  |
| Notes: The number of observations of each index and basic measure is 362, the number of facilities surveyed. Many of the basic measures are based on all the deliveries observed at the facility. This table is meant to show the correlation between the indices generated through principal component analysis and their input variables. The overall index of care quality was created through principal component analysis using the structure, process, and outcomes indices. The process index was the result of principal component analysis using each of the process sub-indices, specifically clinical competence, referral process, antenatal care quality, obstetric care quality, and postnatal care quality. Each of the process sub-indices were generated through principal component analysis using the basic measures listed in this table. | | | | | | | |

**Table A.3.: Correlation Matrix of the Overall Index, Outcome Index, Sub-indices and Basic Measures**

|  | **Overall Index** | **Index** | **Sub-indices/Basic Measures** | | |
| --- | --- | --- | --- | --- | --- |
| **Overall Index** | Care quality | Outcomes | Neo. death | Mat. death | Obs. comp. |
| Overall care quality | 1.00 |  |  |  |  |
|  |  |  |  |  |  |
| **Index** |  |  |  |  |  |
| Outcomes | 0.06 | 1.00 |  |  |  |
|  |  |  |  |  |  |
| **Sub-indices/Basic Measures** |  |  |  |  |  |
| Rate of neonatal deaths (per 1000 deliveries) | -0.04 | -0.73 | 1.00 |  |  |
| Rate of maternal deaths (per 1000 deliveries) | -0.13 | -0.29 | 0.00 | 1.00 |  |
| Rate of obstetric complications (per delivery) | -0.02 | -0.07 | -0.03 | -0.02 | 1.00 |
|  |  |  |  |  |  |
| Notes: The number of observations of each index and basic measure is 362, the number of facilities surveyed. This table is meant to show the correlation between the indices and their input variables. The overall index of care quality was created through principal component analysis using the structure, process, and outcomes indices. The outcome index was created using each of the outcome sub-indices or basic measures listed in this table, specifically the rate of neonatal deaths, the rate of maternal deaths, and the rate of obstetric complications. | | | | | |
