# Supplementary material for: Utilization and quality: How the quality of care influences demand for obstetric care in Nigeria
Source: PLoS One. 2019 Feb 7;14(2):e0211500. doi: 10.1371/journal.pone.0211500 (PMC6366755; doi:10.1371/journal.pone.0211500)
Supplement: S2 File — (PDF) [file pone.0211500.s002.pdf]

Better Obstetrics in Rural Nigeria (BORN) Study
Health Facility Questionnaire

A. Cover Page

Identification Section

1. State Name
2. LGA Name:
3. PHC Name:
4. Community Name:
5. Name of Clinic Officer-in-Charge (OIC):
6. GSM Number of OIC:
7. Is respondent the OIC?
   - € Yes
   - € No
8. Name of respondent: ____
9. Job title of respondent ____
10. GSM Number of respondent: ____
11. Interviewer's Name: ____

B. Facility and Infrastructure

1.	Type of health facility	€ Health post/dispensary<br>€ Primary Health Clinic/Basic Health Clinic<br>€ Primary Health Center<br>€ Comprehensive Health Center<br>€ Other (Specify):
2.	How many beds are available for patients in this facility (total)?	
3.	Is this facility open 24 hours a day/7 days a week (24/7)?	€ Yes<br>€ No
4.	Does this facility have electricity?	€ Yes<br>€ No
5.	What is the primary source of electricity?	€ Electricity (PHCN)<br>€ Generator<br>€ Solar<br>€ Other (Specify):
6.	Is the electricity functioning?	€ Yes<br>€ No
7.	Is a back-up generator available?	€ Yes<br>€ No
8.	Is the generator functional?	€ Yes<br>€ No
9.	In the last month, how many days were you without electricity throughout the day?	
10.	What is the primary source of water?	€ Piped Water<br>€ Borehole with Hand Pump<br>€ Open Well<br>€ Rainwater<br>€ Other (Specify):
11.	What kind of toilet do you have in this facility?	€ Pit Latrine<br>€ VIP<br>€ Water Closet (flush toilet)<br>€ No toilet
12.	Is the toilet functioning?	€ Yes<br>€ No

C. Referral

13.	When referrals of pregnant women or women in labor are made from this clinic, is there communication with the referral hospital e.g. by phone or radio?	€ Yes<br>€ No
14.	When referrals of pregnant women or women in labor are made from this clinic, how often do you communicate with the referral hospital?	€ Always<br>€ Most times<br>€ Sometimes<br>€ Seldom<br>€ Never
15.	How is the communication made?	€ Landline phone<br>€ Clinic cellphone<br>€ Staff cellphone<br>€ 2-way radio<br>€ No means of communication
16.	Is there airtime (credit) on this cell phone at this moment?	€ Yes<br>€ No
17.	Is there a formal mechanism for reimbursing staff for airtime (if they use their personal phones)?	€ Yes<br>€ No
18.	Is there a cell phone signal at this facility?	€ Yes<br>€ No
19.	When an emergency obstetric referral is made how is the patient usually transported to the referral hospital?	€ Clinic ambulance<br>€ Other clinic motor vehicle<br>€ Staff personal vehicle<br>€ Patient vehicle or public transport<br>€ Other (specify)
20.	Does this facility have a motor vehicle ambulance	€ Yes, and functional<br>€ Yes, but not functional<br>€ No
21.	Does this facility have a motorcycle ambulance	€ Yes, and functional<br>€ Yes, but not functional<br>€ No
22.	Does this facility have a bicycle ambulance	€ Yes, and functional<br>€ Yes, but not functional<br>€ No
23.	Does this facility have other form of transportation e.g. animal-drawn cart?	€ Yes (specify)<br>€ No

24.	How far is the nearest referral hospital?	________ km
25.	How long does it take to get to that referral hospital?	______ Hours _________ Minutes
26.	How often is feedback on referred patients given by the referral hospital?	€ Always<br>€ Most times<br>€ Sometimes<br>€ Seldom<br>€ Never
27.	Does this facility provide feedback to the referral hospital?	€ Yes, always<br>€ Most times<br>€ Sometimes<br>€ Seldom<br>€ Never

D. Staffing

	28. Number currently employed	29. Number present today
1. Doctor (non-NYSC)		
2. Doctor (NYSC)		
3. Nurses		
4. Midwives		
5. Nurse midwives		
6. Laboratory scientists		
7. Laboratory technicians/technologists		
8. Radiographers		
9. Pharmacists		
10. Pharmacy technicians and assistants		
11. Health social workers/welfare workers		
12. Nutritionists		
13. Administrators		
14. Medical Records Officers		
15. Public Health Nursing Officers		
16. Environmental Health Officers		
17. Community Health Officers		
18. Community Health Extension Workers (CHEWS)		
19. Junior Community Health Extension Workers (J-CHEWS)		
20. Other Cadre (specify)		
21. Other Cadre (specify)		

Now I am going to ask you about each midwife employed in this facility.

30. Name of midwife	31. Start date in this facility	32. MSS midwife?	Present today?
	DD/MM/YY	€ Yes<br>€ No	€ Yes<br>€ No

E. Provider availability

41.	Is there a doctor physically present in the clinic 24 hours a day/7 days a week?	€ Yes<br>€ No
42.	In the last 7 days, for how many hours was a doctor physically present in the health facility?	__________
43.	Is there a midwife or nurse midwife physically present in the health facility 24 hours a day/7 days a week?	€ Yes<br>€ No
44.	In the last 7 days, for how many hours was a midwife or nurse midwife physically present in the health facility?	__________
45.	Does the facility have a staff register?	€ Yes, seen<br>€ Yes, not seen<br>€ No

F. Service Provision

Basic Services

46.	Does this facility have a laboratory?	€ Yes<br>€ No
47.	Does the lab offer testing for Malaria?	€ Yes<br>€ No
48.	Does the lab offer Hemoglobin Test (Hb or PCV)?	€ Yes<br>€ No
49.	Does the lab offer Urine Test (protein, sugar)?	€ Yes<br>€ No
50.	Does this facility offer antenatal care (ANC) services?	€ Yes<br>€ No
51.	Does this facility offer delivery services?	€ Yes<br>€ No
52.	Are delivery services available 24/7?	€ Yes<br>€ No
53.	Is Caesarean Section provided in this facility?	€ Yes<br>€ No
54.	Is HIV testing offered in this facility?	€ Yes<br>€ No
55.	Are Prevention of mother-to-child transmission of HIV (PMTCT) services available?	€ Yes<br>€ No
56.	Is post-natal care (PNC) provided?	€ Yes<br>€ No
57.	Are family planning services offered in this facility?	€ Yes<br>€ No
58.	Are oral contraceptive pills given?	€ Yes<br>€ No
59.	Is Depoprovera (injectable) given?	€ Yes<br>€ No
60.	Are male condoms given?	€ Yes<br>€ No
61.	Are female condoms given?	€ Yes<br>€ No
62.	Is spermicide given?	€ Yes<br>€ No

63.	Are IUDs (loop) given?	€ Yes<br>€ No
64.	Are implants given?	€ Yes<br>€ No
65.	Is natural family planning (Billing's) counseled?	€ Yes<br>€ No
66.	Is emergency contraception provided?	€ Yes<br>€ No
67.	Are child welfare services offered in this facility?	€ Yes<br>€ No
68.	Is growth monitoring done?	€ Yes<br>€ No
69.	Is routine immunization available?	€ Yes<br>€ No
70.	Is Vitamin A supplementation given?	€ Yes<br>€ No
71.	Is lactation management provided?	€ Yes<br>€ No
72.	Are nutrition services provided?	€ Yes<br>€ No

Emergency Obstetrics Services

73.	Is Manual Vacuum Aspiration (MVA) provided?	€ Yes<br>€ No
74.	Is a functional MVA set available?	€ Yes<br>€ No
75.	Number available	
76.	Is dilation and curettage (D&C) performed?	€ Yes<br>€ No
77.	Is a functional D&C set available?	€ Yes<br>€ No
78.	Number available	
79.	Is a vacuum extraction done in this facility?	€ Yes<br>€ No
80.	Is a functional vacuum available?	€ Yes<br>€ No
81.	Number available	
82.	Is forceps delivery done in this facility?	€ Yes

		€ No
83.	Is breech delivery done in this facility?	€ Yes<br>€ No
84.	Is manual removal of placenta done in this facility?	€ Yes<br>€ No
85.	Does this facility provide treatment for women with sepsis?	€ Yes<br>€ No
86.	Does this facility administer parenteral (IV) antibiotics?	€ Yes<br>€ No
87.	Does this facility treat women with eclampsia?	€ Yes<br>€ No
88.	Does this facility administer parenteral (IV) anticonvulsants?	€ Yes<br>€ No
89.	Does this facility treat women with postpartum hemorrhage?	€ Yes<br>€ No
90.	Does this facility administer uterotonic drugs – parenteral (IV) oxytocics?	€ Yes<br>€ No
91.	Does this facility provide newborn care?	€ Yes<br>€ No
92.	Does this facility resuscitate newborn with bag and mask?	€ Yes<br>€ No
93.	Does this facility provide kangaroo mother care?	€ Yes<br>€ No
94.	Is cord care done at this facility?	€ Yes<br>€ No
95.	Is eye care done at this facility?	€ Yes<br>€ No
96.	Is skin care done at this facility?	€ Yes<br>€ No

G. Drugs, Equipment, and Supplies

97.	Does this facility have a pharmacy?	€ Yes<br>€ No
98.	Does this facility have a supply of medicines?	€ Yes<br>€ No
99.	Is there a drug inventory register?	€ Yes<br>€ No
100.	Is the pharmacy accessible 24 hours a day?	€ Yes<br>€ No
101.	Does this facility have at least one functioning refrigerator?	€ Yes<br>€ No
	Does this facility have ___ in stock?	
102.	Amoxicillin	€ Yes<br>€ No
103.	Ampiclox (injection)	€ Yes<br>€ No
104.	Cefixime	€ Yes<br>€ No
105.	Ceftriaxone	€ Yes<br>€ No
106.	Cefotaxime injection (for newborn)	€ Yes<br>€ No
107.	Erythromicin	€ Yes<br>€ No
108.	Oral flucloxacillin (for newborn)	€ Yes<br>€ No
109.	Gentamicin (injection)	€ Yes<br>€ No
110.	Metronidazole (injection)	€ Yes<br>€ No
111.	Penicillin G (Benzyl)	€ Yes<br>€ No
112.	Trimethoprim/sulfamethoxazole	€ Yes<br>€ No
113.	Tetracycline eye ointment/drops	€ Yes<br>€ No

114.	Magnesium sulfate (injection)	€ Yes<br>€ No
115.	Diazepam (injection)	€ Yes<br>€ No
116.	Hydralazine	€ Yes<br>€ No
117.	Labetalol	€ Yes<br>€ No
118.	Methyldopa	€ Yes<br>€ No
119.	Nifedipine	€ Yes<br>€ No
120.	Ergometrine (injection)	€ Yes<br>€ No
121.	Methylergometrine (injection)	€ Yes<br>€ No
122.	Oxytocin (injection)	€ Yes<br>€ No
123.	Prostaglandin E2 (dinoprostone)	€ Yes<br>€ No
124.	Adrenaline (epinephrine)	€ Yes<br>€ No
125.	Aminophylline	€ Yes<br>€ No
126.	Diphenhydramine	€ Yes<br>€ No
127.	Hydrocortisone	€ Yes<br>€ No
128.	Promethazine	€ Yes<br>€ No
129.	Chloroquine	€ Yes<br>€ No
130.	Quinine Dihydrochloride	€ Yes<br>€ No
131.	Artemisium–based combination therapy (ACT)	€ Yes<br>€ No
132.	Sulphadoxine/pyrimethamine (SP)	€ Yes<br>€ No

133.	Combined oral contraceptives	€ Yes<br>€ No
134.	3-month injectables	€ Yes<br>€ No
135.	Intrauterine devices (IUDs)	€ Yes<br>€ No
136.	Male condoms	€ Yes<br>€ No
137.	Female condoms	€ Yes<br>€ No
138.	Emergency contraception	€ Yes<br>€ No
139.	Misoprostol tablets	€ Yes<br>€ No
140.	IV Solutions	€ Yes<br>€ No
141.	Vitamin K (for newborn)	€ Yes<br>€ No
142.	Oral rehydration solution (ORS)	€ Yes<br>€ No
143.	Ferrous sulfate or fumarate	€ Yes<br>€ No
144.	Folic acid	€ Yes<br>€ No
145.	Tetanus toxoid vaccine	€ Yes<br>€ No
146.	Anti-Rho (D) immune globulin	€ Yes<br>€ No
147.	Antishock garments	€ Yes<br>€ No

Are there written guidelines or protocols available for…?		
148.	Management of obstetric and newborn complications	€ Yes, seen<br>€ Yes, not seen<br>€ No
149.	Immediate newborn care	€ Yes, seen<br>€ Yes, not seen<br>€ No
150.	Kangaroo mother care	€ Yes, seen<br>€ Yes, not seen<br>€ No
151.	Antenatal care	€ Yes, seen<br>€ Yes, not seen<br>€ No
152.	Prevention of mother-to-child transmission of HIV (PMTCT) (maternal and newborn dosing)	€ Yes, seen<br>€ Yes, not seen<br>€ No
153.	Infection prevention for HIV/AIDS (universal precautions)	€ Yes, seen<br>€ Yes, not seen<br>€ No
154.	Safe abortion	€ Yes, seen<br>€ Yes, not seen<br>€ No
155.	Post-abortion care	€ Yes, seen<br>€ Yes, not seen<br>€ No
156.	Family planning	€ Yes, seen<br>€ Yes, not seen<br>€ No

		Is item available?	How many are functional?
157.	Sphygmomanometer (BP cuff)	€ Yes<br>€ No	
158.	Stethoscope	€ Yes<br>€ No	

159.	Fetal stethoscope	€ Yes<br>€ No	
160.	Adult weighing scale	€ Yes<br>€ No	
161.	Baby weighing scale	€ Yes<br>€ No	
162.	Examination couch	€ Yes<br>€ No	
163.	Angle poised lamp	€ Yes<br>€ No	
164.	Delivery bed	€ Yes<br>€ No	
165.	Sterilizer or autoclave	€ Yes<br>€ No	
166.	Wheel Chairs	€ Yes<br>€ No	
167.	Midwifery (Mama) Kits	€ Yes<br>€ No	
168.	Oxygen/Resuscitation Set	€ Yes<br>€ No	
169.	Clinical oral thermometer	€ Yes<br>€ No	
170.	Rectal thermometer for newborn	€ Yes<br>€ No	
171.	IV Infusion stand(s)	€ Yes<br>€ No	
172.	Newborn resuscitation table	€ Yes<br>€ No	
173.	Incubator	€ Yes<br>€ No	
174.	Radiant warmer	€ Yes<br>€ No	
175.	Delivery Set/Pack	€ Yes<br>€ No	
176.	Vacuum extractor with different size cups	€ Yes<br>€ No	
177.	Neonatal Mucus extractor	€ Yes<br>€ No	

178.	Ambu (ventilatory) bag	€ Yes<br>€ No	
179.	Suction aspirator (operated by foot or electrically)	€ Yes<br>€ No	

H. Health Management Information System (HMIS)

Are the following registers available in your health facility?

180.	Delivery register	€ Yes – official<br>€ Yes – Improvised<br>€ No
181.	Antenatal register or tally sheet	€ Yes – official<br>€ Yes – Improvised<br>€ No
182.	Postnatal register or tally sheet	€ Yes – official<br>€ Yes – Improvised<br>€ No
183.	Newborn Register	€ Yes – official<br>€ Yes – Improvised<br>€ No
184.	Family planning register or tally sheet	€ Yes – official<br>€ Yes – Improvised<br>€ No
185.	Under- five clinic register or tally sheet	€ Yes – official<br>€ Yes – Improvised<br>€ No
186.	Immunization register or tally sheet	€ Yes – official<br>€ Yes – Improvised<br>€ No
187.	Outpatient register	€ Yes – official<br>€ Yes – Improvised<br>€ No
188.	Inpatient Register	€ Yes – official<br>€ Yes – Improvised<br>€ No
189.	Discharge Summary	€ Yes – official<br>€ Yes – Improvised<br>€ No

CHECK REGISTERS AND COLLECT INFORMATION FROM JULY TO DECEMBER 2013.

		Jul	Aug	Sept	Oct	Nov	Dec
190.	Number of new antenatal (ANC) clients (first visit)						
191.	Number of ANC re-visits						
192.	Total number of ANC visits						
193.	Total number of women that received at least 2+ doses of tetanus toxoid						
194.	Number of deliveries in health facility						
195.	Number of live births						
196.	Number of still births						
197.	Number of early neonatal deaths (within 1 week of life)						
198.	Number of deaths of babies aged 0-28 days						
199.	Total number of maternal deaths in the last 12 months						
200.	Number of breech deliveries						
201.	Number of obstetric emergencies seen						
202.	Number of postpartum hemorrhage cases seen						
203.	Number of cases with retained placenta						
204.	Number of cases with prolonged/obstructed labor						
205.	Number of cases with ruptured uterus						
206.	Number of cases with postpartum sepsis						
207.	Number of cases with severe pre-eclampsia/eclampsia						
208.	Number of cases with abortion complications (hemorrhage and/or sepsis)						
209.	Number of vacuum extractions performed						
210.	Number of MVAs carried out						
211.	Number of manual placenta removals carried out						
212.	Number of obstetric clients referred out due to emergency obstetric complications						
213.	Number of sick newborn babies referred out for further treatment						
214.	Number of post-natal reviews within 48 hours						
215.	Number of post-natal visits in first 2 weeks						
216.	Number of post-natal visits at 6 weeks						

217.	Number of visits for growth monitoring (children <1 year of age)						
218.	Number of children < 1 year of age immunized with BCG						
219.	Number of children < 1 year of age immunized with DPT-1						
220.	Number of children < 1 year of age immunized with polio-1						
221.	Number of children < 1 year of age immunized with DPT-3						
222.	Number of children < 1 year of age immunized with polio-3						
223.	Number of children< 1 year of age immunized against measles						
224.	Number of newborns who received treatment						
225.	Number of sick under-five children who received consultations services						
226.	Number of new family planning clients (first visit)						
227.	Number of family planning clients (subsequent visits)						

For the last five obstetric referrals in the health facility, collect the following information

228.				
		**Date**	**Provisional Diagnosis or Reason for Referral**	**Age**
	1.			
	2.			
	3.			
	4.			
	5.			

I. General

229.	In an obstetric emergency, is payment required before a woman can receive treatment in this facility?	€ Yes<br>€ No
	How much does this facility charge for:	
230.	ANC registration	
231.	Subsequent ANC visit	
232.	Normal delivery	______
233.	Admission	______
234.	Instrumental delivery with vacuum	______
235.	Instrumental delivery with forceps	______
236.	Is there a formal system in place to have fees for maternity services waived for poor women?	€ Yes<br>€ No
237.	Is there an informal system in place to have fees for maternity services waived for poor women?	€ Yes<br>€ No

J: Health Worker Assessment

238.	Sex	€ Male<br>€ Female
239.	Date of Birth	DD/MM/YY<br>€ Unknown
240.	What is your estimated age?	
241.	What is your current professional qualification?	€ Medical degree (MBBS)<br>€ Community Health Officer certificate<br>€ Nursing certificate<br>€ Midwife certificate<br>€ Nurse/Midwife certificate<br>€ CHEW certificate<br>€ Junior CHEW certificate<br>€ Other (specify)
242.	In what year did you graduate with this qualification?	
243.	How many years of schooling have you completed in total (including primary, secondary, and any further education)?	
244.	When did you start working in this health facility?	DD/MM/YY
245.	Are you employed by the Midwives Service Scheme?	€ Yes<br>€ No
246.	What was your status before you enrolled in the Midwives Services Scheme (MSS)?	€ Student<br>€ Unemployed<br>€ Retired<br>€ Other (specify)
247.	What were your reasons for enrollment in the Midwives Service Scheme (MSS)?	€ Salary<br>€ Employment Benefits<br>€ Personal fulfillment and voluntary service<br>€ Other (Specify):
248.	How much do you earn in total per month through the MSS (from federal, state, and local government sources)?	N __________
249.	Within the last 12 months, how many months was your federal government salary not paid on time?	___________
250.	Are you currently owed any federal government salary arrears?	€ Yes<br>€ No
251.	How many months are you owed?	____________

252.	Within the last 12 months, how many months was your state government salary not paid on time?	
253.	Are you currently owed any state government salary arrears?	€ Yes<br>€ No
254.	How many months are you owed?	
255.	Is accommodation provided for you through the MSS?	€ Yes<br>€ No
256.	What kind of accommodation is provided?	€ Single room<br>€ Room and parlor<br>€ Flat<br>€ Bungalow<br>€ Other (specify)
257.	How many bedrooms do you have?	
258.	Do you have to pay rent for this accommodation?	€ Yes<br>€ No
259.	How much do you pay per month for this accommodation?	N ___________
260.	How much does it normally cost in this community to rent an accommodation like this?	N ___________ per month
261.	In an average week, how many hours do you work in this health facility?	
262.	When was the last time you took a delivery?	MM/YY<br>€ Never
263.	Within the last 6 months, how many deliveries in total would you say you have taken?	_______
264.	Among the various things related to your working situation that you would like to see improved, can you tell me the three most important? Please rank them in order of importance, with 1 being the most important.	_____ More support from supervisor<br>_____ More knowledge/updates training<br>_____ More supplies/stock<br>_____ Better quality equipment/supplies<br>_____ Less workload (more staff)<br>_____ Better working hours/flexible times<br>_____ Higher salary<br>_____ Other benefits<br>_____ Increased security<br>_____ Better facility infrastructure<br>_____ More autonomy/independence<br>_____ Emotional support for staff (counseling/social activities)<br>_____ Other

**Have you received a training course in ____ post qualification?**		
265.	Emergency Obstetric Care	€ Yes

		€ No
266.	Essential Newborn Care	€ Yes<br>€ No
267.	Neonatal Resuscitation	€ Yes<br>€ No
268.	Pregnancy childbirth and postnatal care	€ Yes<br>€ No
269.	Partograph training	€ Yes<br>€ No
270.	WHO new model of ANC (Focused antenatal care)	€ Yes<br>€ No
271.	Family Planning	€ Yes<br>€ No
272.	Newborn/Infant Feeding	€ Yes<br>€ No
273.	Integrated Management of Childhood Illnesses (IMCI)	€ Yes<br>€ No
274.	HIV/AIDS counseling and testing	€ Yes<br>€ No
275.	Integrated Prevention of mother to child transmission (PMTCT) of HIV	€ Yes<br>€ No

How satisfied are you with the following aspects of your work?

276.	Your working relationship with other facility staff	€ Very unsatisfied<br>€ Unsatisfied<br>€ Neither satisfied nor unsatisfied<br>€ Satisfied<br>€ Very satisfied
277.	Your relationship with local traditional rulers	€ Very unsatisfied<br>€ Unsatisfied<br>€ Neither satisfied nor unsatisfied<br>€ Satisfied<br>€ Very satisfied
278.	Your relationship with Ward Development Committee	€ Very unsatisfied<br>€ Unsatisfied<br>€ Neither satisfied nor unsatisfied<br>€ Satisfied<br>€ Very satisfied

279.	The availability of medicines in the facility	€ Very unsatisfied<br>€ Unsatisfied<br>€ Neither satisfied nor unsatisfied<br>€ Satisfied<br>€ Very satisfied
280.	The physical condition of the health facility	€ Very unsatisfied<br>€ Unsatisfied<br>€ Neither satisfied nor unsatisfied<br>€ Satisfied<br>€ Very satisfied
281.	Your ability to provide high quality service	€ Very unsatisfied<br>€ Unsatisfied<br>€ Neither satisfied nor unsatisfied<br>€ Satisfied<br>€ Very satisfied
282.	Your respect in the community	€ Very unsatisfied<br>€ Unsatisfied<br>€ Neither satisfied nor unsatisfied<br>€ Satisfied<br>€ Very satisfied
283.	Your training opportunities to upgrade your skills	€ Very unsatisfied<br>€ Unsatisfied<br>€ Neither satisfied nor unsatisfied<br>€ Satisfied<br>€ Very satisfied
284.	Your ability to meet the needs of the community	€ Very unsatisfied<br>€ Unsatisfied<br>€ Neither satisfied nor unsatisfied<br>€ Satisfied<br>€ Very satisfied
285.	Your salary	€ Very unsatisfied<br>€ Unsatisfied<br>€ Neither satisfied nor unsatisfied<br>€ Satisfied<br>€ Very satisfied
286.	Your employment benefits (transport, health insurance, bonus etc.)	€ Very unsatisfied<br>€ Unsatisfied<br>€ Neither satisfied nor unsatisfied<br>€ Satisfied<br>€ Very satisfied

287.	Your safety and security to live and practice in this community	€ Very unsatisfied<br>€ Unsatisfied<br>€ Neither satisfied nor unsatisfied<br>€ Satisfied<br>€ Very satisfied
288.	Your living accommodation	€ Very unsatisfied<br>€ Unsatisfied<br>€ Neither satisfied nor unsatisfied<br>€ Satisfied<br>€ Very satisfied
289.	Recognition of your work by your superiors	€ Very unsatisfied<br>€ Unsatisfied<br>€ Neither satisfied nor unsatisfied<br>€ Satisfied<br>€ Very satisfied
290.	How would you rate overall satisfaction with your job	€ Very unsatisfied<br>€ Unsatisfied<br>€ Neither satisfied nor unsatisfied<br>€ Satisfied<br>€ Very satisfied

Now I would like you to answer some questions related to maternal and newborn care.

Directions: Read the following questions and select the single best answer to each question.

291.	Pregnant women should receive educational messages about which of the following?	€ Personal hygiene, rest, and exercise during pregnancy<br>€ Diet and nutrition during pregnancy<br>€ Danger signs during pregnancy<br>€ All of the above
292.	When counseling a pregnant woman about nutrition, be sure to	€ Ask her what she eats in a typical day to determine if her diet is adequate<br>€ Tell her to eat the same amount of food that she ate before her pregnancy<br>€ Recommend that she weigh herself once a week<br>€ Inform her that only very anemic women need iron/folate supplements
293.	Focused antenatal care includes which of the following actions?	€ Checking the baby's position at 28 weeks<br>€ Checking the woman's blood pressure at every visit<br>€ Assessing ankle edema at 36 weeks<br>€ Counseling the woman about danger signs only at the last visit
294.	Tests that should be performed for every woman during antenatal care include	A. Hemoglobin<br>B. Test for syphilis<br>C. Ultrasound of baby<br>D. A and B only
295.	When performing a vaginal examination, which of the following is recorded on the partograph?	€ Cervical dilation of 3 centimeters<br>€ Vaginal temperature and wetness<br>€ Position of the presenting part<br>€ Degree of molding
296.	Active management of the third stage of labor should be practiced	€ Only for women who have a history of postpartum hemorrhage<br>€ Only for the primipara<br>€ Only for the multipara<br>€ For all women in labor

297.	The appropriate order of steps in active management of the third stage of labor include	€ Controlled cord traction, fundal massage, and oxytocin<br>€ Intravenous oxytocin, cord clamping and cutting, and fundal massage<br>€ Cord clamping and cutting, controlled cord traction, ergometrine administration, and inspection to be sure placenta is intact<br>€ Intramuscular injection of oxytocin, controlled cord traction with countertraction to the uterus, and uterine massage
298.	Which of the following will help to decrease the risk of infection during childbirth?	€ Performing frequent vaginal examinations<br>€ Rupturing membranes as soon as possible in the first stage of labor<br>€ Routing catheterization of the bladder before childbirth<br>€ Reducing prolonged labor
299.	Immediate care for a normal newborn includes	€ Skin-to-skin contact followed by placing the baby in a warming incubator<br>€ Drying the baby, removing the wet cloth, and covering the baby with a clean, dry cloth<br>€ Stimulating the baby by slapping the soles of the baby's feet<br>€ Deep suctioning of the airway to remove mucus
300.	To maintain the newborn's axillary temperature between 36.6° C and 37.5° C, it is important to	€ Place the baby in an incubator<br>€ Bathe the baby in warm water immediately after birth<br>€ Rub the baby vigorously with a blanket<br>€ Cover the baby's head, place the baby in skin-to-skin contact on the mother's chest, and cover with a blanket
301.	The best way to determine if a newborn needs resuscitation is to	€ Wait until 1 minute after birth and assign the Apgar score<br>€ Listen to the baby's heart rate<br>€ Observe respirations immediately and begin resuscitation if they are less than 30/minute<br>€ Perform resuscitation only if central cyanosis is present
302.	When counseling the mother about breastfeeding, the skilled provider should tell her to	€ Avoid giving colostrum to the newborn<br>€ Establish a schedule for breastfeeding so the baby gets plenty of sleep<br>€ Give the baby water after each feed<br>€ Breastfeed on demand for as long as the baby wants to feed

303.	Immediate postpartum hemorrhage can be due to	€ Uterine atony<br>€ Genital trauma<br>€ Retained placenta<br>€ All of the above
304.	The most effective way to immediately control eclamptic convulsions is to	€ Give diazepam<br>€ Give magnesium sulfate<br>€ Deliver the baby as soon as possible<br>€ Give nifedipine
305.	Do not perform vacuum extraction in the case of	€ A cephalic presentation<br>€ A face presentation<br>€ Cervical dilation of 7cm<br>€ Fetal head not engaged
306.	A woman with a ruptured uterus has which of the following signs and symptoms	€ Rapid maternal pulse<br>€ Persistent abdominal pain and suprapubic tenderness<br>€ Fetal distress<br>€ All of the above
307.	During the first 2 hours following birth, the provider should	€ Measure the woman's blood pressure and pulse once, and insert a catheter to empty her bladder<br>€ Measure the woman's blood pressure and pulse, and check the uterine tone every 15 minutes<br>€ Not disturb the woman if asleep because her rest is more important than her vital signs<br>€ Measure the woman's temperature and pulse, massage the uterus, and perform a vaginal examination to remove clots
308.	After childbirth, the mother should have a postpartum visit with a skilled provider	€ Once, at 3 weeks postpartum<br>€ Once, at 6 weeks postpartum<br>€ Three times: At 6 hours, 6 days, and 6 weeks postpartum and any time she has danger signs<br>€ Only if she has danger signs
309.	During each postpartum visit, specific information should be obtained from the woman about	€ Problems during pregnancy, during and after childbirth, and any present problems<br>€ Present problems only<br>€ Only those problems directly related to childbirth<br>€ None of the above

<table>
<tr><td>310.</td><td>When counseling a new mother about breastfeeding in the 6 hours following birth</td><td>€ Help her position the baby so that he/she attaches properly to the nipple
€ Tell her to give breast milk substitutes so her baby will grow faster
€ Advise that she breastfeed her baby 4 times/day
€ Tell her that she needs a method of contraception even if she is exclusively breastfeeding</td></tr>
</table>

CASE STUDY

<table>
<tr><td colspan="3">Mrs. B is a 30-year-old gravida 4, para 4. She gave birth at the health center to a healthy, full-term baby weighing 4.2kg. You gave oxytocin 10 units IM following birth of the baby. The placenta was delivered 5 minutes later without complication. However, 30 minutes after childbirth, Mrs. B tells you that she is having heavy vaginal bleeding.</td></tr>
<tr><td>311.</td><td>What is the first action you will take?</td><td>€ Check the uterus to see whether it is contracted
€ Administer more oxytocin
€ Perform bimanual compression of the uterus
€ Perform manual exploration of the uterus</td></tr>
<tr><td>312.</td><td>Vaginal bleeding immediately after birth is the presence of a well contracted uterus is most often due to</td><td>€ Uterine atony
€ Endometritis
€ Genital trauma
€ Abnormal clotting mechanism</td></tr>
<tr><td colspan="3">You have completed your assessment of Mrs. B and your main findings include the following
• Pulse 88/minute
• Respiration rate is 18/minute
• Blood pressure 110/80
• Temperature 37°C
Her uterus is firm and well contracted. The placenta is complete. She has no perineal trauma. It is difficult to examine the vagina and cervix because she continues to have heavy vaginal bleeding.</td></tr>
<tr><td>313.</td><td>Based on these findings, what is your next step?</td><td>€ Pack the uterus and the vagina
€ Begin a blood transfusion
€ Start antibiotics
€ Perform speculum examination of the vagina to identify and repair tears</td></tr>
</table>

314.	What will you tell your assistant to do while you perform the exam?	€ Monitor vital signs and begin intravenous fluids<br>€ Reassure Mrs. B and her family<br>€ Draw blood for hemoglobin<br>€ All of the above
One hour following childbirth, you repair Mrs. B's cervical tear. After repair of the cervical tear, Mrs. B's hemoglobin is found to be 10g/dL, and her vital signs are stable.		
315.	What is the most appropriate plan of care?	€ Begin transfusing blood<br>€ Send her home<br>€ Monitor her vital signs for 24 hours and begin ferrous sulphate and folate supplementation; encourage breastfeeding<br>€ Continue administration of oxytocin for 24 hours

K. Observation

316.	What is the state of the clinic building(s)?	€ Good (requires no rehabilitation)<br>€ Fair (requires minor rehabilitation)<br>€ Poor (requires major rehabilitation)
317.	Is there functional fan/air conditioning in the delivery room?	€ Yes<br>€ No
318.	Are there curtains/means of providing patient privacy?	€ Yes<br>€ No
319.	Is there a waiting area for visitors and family?	€ Yes<br>€ No
