# Supplementary material for: Utilization and quality: How the quality of care influences demand for obstetric care in Nigeria
Source: PLoS One. 2019 Feb 7;14(2):e0211500. doi: 10.1371/journal.pone.0211500 (PMC6366755; doi:10.1371/journal.pone.0211500)
Supplement: S3 File — (PDF) [file pone.0211500.s003.pdf]

Better Obstetrics in Rural Nigeria (BORN) Study
Household Questionnaire

A. Cover Page

Identification Section

1. State Name:
2. LGA Name:
3. PHC Name:
4. Interviewer Name:

5. Is a competent respondent present in the selected household?
   - € Yes
   - € No
6. Is the entire household away for an extended period?
   a. Yes
   b. No

ADMINISTER CONSENT

7. Consent given?
   - € Yes
   - € No

B. Household Roster

	Name	Who is providing the information?
1. HH Head		☐
2.		☐
3.		☐
4.		☐
5.		☐
6.		☐
7.		☐
8.		☐
9.		☐
10.		☐
11.		☐
12.		☐
13.		☐
14.		☐
15.		☐
16.		☐
17.		☐
18.		☐
19.		☐
20.		☐
21.		☐
22.		☐
23.		☐
24.		☐
25.		☐
26.		☐
27.		☐
28.		☐
29.		☐
30.		☐

1.	What is NAME's relationship to the head of the household?	€ Head of household<br>€ Spouse (wife/husband)<br>€ Own son / daughter<br>€ Step son/daughter<br>€ Son/daughter in-law<br>€ Grandchild<br>€ Brother/sister<br>€ Parent<br>€ Parent in law<br>€ Niece/nephew<br>€ Other relative<br>€ Domestic help<br>€ Other non-relative<br>€ Co-wife<br>€ Other (Specify):
2.	What is NAME's date of birth?	Month/Day/Year<br>€ Unknown
3.	What is NAME's estimated age?	
4.	What is NAME's current marital status?	a. Never married<br>b. Monogamously married<br>c. Polygamously married<br>d. Civil union<br>e. Divorced/Separated<br>f. Widowed
5.	Is NAME male or female?	€ Male<br>€ Female
6.	Has NAME been pregnant at any time since January 2009 regardless of the outcome of the pregnancy?	€ Yes<br>€ No
7.	Is NAME'S natural mother alive?	€ Yes<br>€ No
8.	Does NAME'S mother usually live in the household?	€ Yes<br>€ No
9.	Is NAME'S natural father alive?	€ Yes<br>€ No
10.	Does NAME'S natural father usually live in this household?	€ Yes<br>€ No

11.	Has NAME ever attended school?	€ Yes<br>€ No
12.	Highest level of schooling attained	1. Pre-Primary/Kindergarten<br>€ One<br>€ Two<br>€ Three<br>2. Primary School<br>€ One<br>€ Two<br>€ Three<br>€ Four<br>€ Five<br>€ Six<br>3. Secondary School<br>€ One<br>€ Two<br>€ Three<br>€ Four<br>€ Five<br>€ Six<br>4. Lower Six<br>5. Upper Six<br>6. NCE / OND / 'AL' level<br>7. BA/BSC/HND<br>8. Masters or higher<br>9. Attended Koranic School
13.	Is NAME currently going to school	€ Yes<br>€ No

C. Household Characteristics

14.	What is the main source of drinking water for members of your household?	Piped Water<br>€ Piped into dwelling<br>€ Piped into yard/plot<br>€ Public tap/standpipe<br>€ Tube well or borehole<br>Dug Well<br>€ Protected well<br>€ Unprotected well<br>Water from Spring<br>€ Protected spring<br>€ Unprotected spring<br>Rainwater<br>Tanker truck<br>Cart with small tank<br>Surface water (river/pond/stream)<br>Bottled water<br>Other (Specify):
15.	What kind of toilet facility do members of your household usually use?	Flush or pour flush toilet<br>Pit latrine<br>€ Ventilated improved Pit latrine<br>€ Pit latrine with slab<br>€ Pit latrine without slab/Open pit<br>Composting toilet<br>Bucket toilet<br>Hanging toilet/hanging Latrine<br>No facility/bush/field<br>Other (Specify):
16.	Do you share this toilet facility with other households?	€ Yes<br>€ No
17.	How many households including yours use this toilet facility?	____<br>€ Don’t know

18.	What type of fuel does your household mainly use for cooking?	€ Electricity<br>€ LPG<br>€ Natural gas<br>€ Biogas<br>€ Kerosene<br>€ Coal, lignite<br>€ Charcoal<br>€ Wood<br>€ Straw/shrubs/grass<br>€ Agricultural crop<br>€ Animal dung<br>€ No food cooked in household<br>€ Other (Specify):
19.	Do you have a separate room that is used as a kitchen?	€ Yes<br>€ No
20.	Main material for the finish of the floor	Natural floor<br>€ Earth/sand<br>€ Dung<br>Rudimentary floor<br>€ Wood planks<br>€ Palm/bamboo<br>Finished floor<br>€ Parquet or polished wood<br>€ Vinyl or asphalt strips<br>€ Ceramic tiles<br>€ Cement<br>€ Carpet/rug<br>€ Other (Specify):
21.	Main material for finish of the roof	Natural roofing<br>€ No roof<br>€ Thatch/palm leaf<br>Rudimentary roofing<br>€ Rustic mat<br>€ Palm/bamboo<br>€ Wood planks<br>€ Cardboard<br>Finished roofing<br>€ Metal/zinc<br>€ Wood<br>€ Ceramic tiles<br>€ Cement<br>€ Roofing shingles<br>Other (Specify):

22.	Main material for finish of the exterior	Natural walls<br>€ No walls<br>€ Cane/palm/trunks<br>€ Dirt (mud)<br>Rudimentary walls<br>€ Bamboo with mud<br>€ Stone with mud<br>€ Plywood<br>€ Cardboard<br>€ Reused wood<br>Finished walls<br>€ Cement<br>€ Stone with lime/cement<br>€ Bricks<br>€ Cement blocks<br>€ Wood planks/shingles<br>Other (Specify):
23.	How many rooms in total are in your household, including rooms for sleeping and all other rooms?	
24.	How many rooms are used for sleeping?	
25.	Does your household have electricity?	€ Yes<br>€ No
26.	Does your household have the following items which are in good working order?	a. A radio<br>b. A television<br>c. A mobile telephone<br>d. A landline telephone<br>e. A refrigerator<br>f. A cable TV<br>g. A generating set<br>h. Air conditioner<br>i. A computer<br>j. Electric iron<br>k. A fan
27.	Does any member of this household own	€ A canoe<br>€ A bicycle<br>€ A motorcycle or motor scooter<br>€ An animal-drawn cart<br>€ A car or truck<br>€ A boat with a motor
28.	Does any member of this household own any agricultural land?	€ Yes<br>€ No

29.	Does any member of this household have a bank account?	€ Yes<br>€ No

D. Mortality

30.	Have there been any deaths in this household within the last 10 years?	€ Yes<br>€ No
31.	How many deaths?	
32.	When did he/she die?	Month/Day/Year
33.	How old was he/she when they died?	______ YEARS (IF OLDER THAN ONE YEAR)<br>______ MONTHS (IF LESS THAN ONE YEAR)<br>______ DAYS (IF 1 MONTH OR LESS)
34.	What was his/her relationship to the head of the household?	€ Head of household<br>€ Spouse (wife/husband)<br>€ Own son / daughter<br>€ Step son/daughter<br>€ Son/daughter in-law<br>€ Grandchild<br>€ Brother/sister<br>€ Parent<br>€ Parent in law<br>€ Niece/nephew<br>€ Other relative<br>€ Domestic help<br>€ Other non-relative<br>€ Co-wife<br>€ Other ______________
35.	Sex of individual	€ Male<br>€ Female
36.	Was NAME pregnant when she died?	€ Yes<br>€ No
37.	Did she die during childbirth?	€ Yes<br>€ No
38.	Did she die within two months after the end of a pregnancy or childbirth?	€ Yes<br>€ No

39.	Where did she give birth?	€ Your home<br>€ Other home<br>€ Govt. Hospital<br>€ (Enter PHC name from Q.3)<br>€ Other Primary Health Care Center (PHC)<br>€ Other public health facility<br>€ Private Hospital/clinic<br>€ Maternity/Nursing home<br>€ Other ________

SECTION E ONWARDS IS FOR FOR ALL WOMEN IN THE HOUSEHOLD WHO REPORTED BEING PREGNANT BETWEEN JANUARY 2009 AND THE DATE OF INTERVIEW

E. Respondent Characteristics

40.	What is your date of birth?	DD/MM/YY<br>€ Unknown
41.	What is your estimated age?	
42.	What is your ethnic group or tribe?	€ Fulani<br>€ Hausa<br>€ Igbo<br>€ Yoruba<br>Other (Specify):
43.	What is your religion?	€ Catholic<br>€ Anglican<br>€ Born-again Christian<br>€ Moslem<br>€ Traditionalist<br>€ Other (Specify):
44.	How long have you been living in your current place of residence?	Years ___ Months ____
45.	I would like you to read this sentence to me: "***The man goes to his farm every day***."	€ Cannot read at all<br>€ Able to read only parts of sentence<br>€ Able to read whole sentence<br>€ Blind/visually impaired
46.	Aside from your own housework, have you done any work in the last seven days?	€ Yes<br>€ No
47.	As you know, some women take up jobs for which they are paid in cash or kind. Others sell things, have a small business or work on the family farm or in the family business. In the last seven days, have you done any of these things or any other work?	€ Yes<br>€ No
48.	Although you did not work in the last seven days, do you have any job or business from which you were absent for leave, illness, vacation, maternity leave or any other such reason?	€ Yes<br>€ No
49.	Have you done any work in the last 12 months?	€ Yes<br>€ No

50.	What is your occupation, that is, what kind of work do you mainly do?	€ Farming<br>€ Laboring/building<br>€ Fishing<br>€ Office job<br>€ Trading/Business<br>€ Teacher<br>€ Soldier/Policeman/Security guard<br>€ Mechanic<br>€ Bar/restaurant attendant<br>€ House maid/Gardener<br>€ Driver/Conductor<br>€ Vendor<br>€ Doctor/Nurse/Healthcare professional<br>€ Student<br>€ Okada (motorcycle)<br>€ Other, specify
51.	Do you do this work for a member of your family, for someone else, or are you self-employed?	€ Family member<br>€ Someone else<br>€ Self-employed
52.	Do you usually work throughout the year, or do you work seasonally, or only once in a while?	€ Throughout the year<br>€ Seasonally/Part of the year<br>€ Once in a while
53.	Are you paid in cash or kind for this work or are you not paid at all?	€ Cash only<br>€ Cash and Kind<br>€ Kind only<br>€ Not paid
54.	How much are you paid for this work?	N________________<br>€ Daily<br>€ Weekly<br>€ Monthly<br>€ Other
55.	Aside from his own housework, has your spouse/partner done any work in the last seven days?	€ Yes<br>€ No
56.	Although he did not work in the last seven days, did he have any job or business from which he was absent for leave, illness, vacation, or any other such reason?	€ Yes<br>€ No
57.	Has he done any work in the last 12 months?	€ Yes<br>€ No

58.	What is his occupation, that is, what kind of work does he mainly do?	€ Farming<br>€ Laboring/building<br>€ Fishing<br>€ Office job<br>€ Trading/Business<br>€ Teacher<br>€ Soldier/Policeman/Security guard<br>€ Mechanic<br>€ Bar/restaurant attendant<br>€ House maid/Gardener<br>€ Driver/Conductor<br>€ Vendor<br>€ Doctor/Nurse/Healthcare professional<br>€ Student<br>€ Okada (motorcycle)<br>€ Other, specify
59.	Does he usually work throughout the year, or does he work seasonally, or only once in a while?	€ Throughout the year<br>€ Seasonally/Part of the year<br>€ Once in a while
60.	Who usually decides how the money you earn will be used: mainly you, mainly your husband/partner, or you and your husband/partner jointly?	€ Respondent<br>€ Husband/partner<br>€ Respondent and husband/partner jointly<br>€ Someone else<br>€ Other
61.	Would you say that the money that you earn is more than what your husband/partner earns, less than what he earns, or about the same?	€ More<br>€ Less<br>€ About the same<br>€ Don’t know
62.	Who usually makes decisions about health care for yourself: you, your husband/partner, you and your husband/partner jointly, or someone else?	€ Respondent<br>€ Husband/partner<br>€ Respondent and husband/partner jointly<br>€ Someone else<br>€ Other

F. Contraception

63.	Would you like to have another child, or would you prefer not to have any (more) children?	€ Have another child<br>€ No more children<br>€ Can't get pregnant<br>€ Undecided/don't know
64.	Comparing with you, do you think your partner wants more children, fewer children, or the same number of children?	€ Same<br>€ More<br>€ Fewer
65.	Do you approve or disapprove of couples using contraceptive methods to avoid getting pregnant?	€ Approve<br>€ Disapprove<br>€ Neither Approve nor Disapprove
66.	Which contraceptive methods have you heard of?	€ Pill<br>€ IUD<br>€ Injectables<br>€ Implants<br>€ Foam or jelly<br>€ Diaphragm<br>€ Female condom<br>€ Male condom<br>€ Female sterilization<br>€ Male sterilization<br>€ Lactational amenorrhea method<br>€ Rhythm / natural method<br>€ Withdrawal<br>€ Other modern method, specify<br>€ Other traditional method (Specify):
67.	Have you ever used…	€ Pill<br>€ IUD<br>€ Injectables<br>€ Implants<br>€ Foam or jelly<br>€ Diaphragm<br>€ Female condom<br>€ Male condom<br>€ Female sterilization<br>€ Male sterilization<br>€ Lactational amenorrhea method<br>€ Rhythm / natural method<br>€ Withdrawal<br>€ Other modern method, specify<br>€ Other traditional method (Specify):

68.	Are you currently doing something or using any method to delay or avoid getting pregnant?	€ Yes<br>€ No
69.	Which method are you using to prevent unwanted pregnancies?	€ Pill<br>€ IUD<br>€ Injectables e.g. depoprovera<br>€ Implants e.g. norplant<br>€ Foam or jelly<br>€ Diaphragm<br>€ Female condom<br>€ Male condom<br>€ Female sterilization<br>€ Male sterilization<br>€ Lactational amenorrhea method<br>€ Rhythm / natural method<br>€ Withdrawal<br>€ Other modern method, specify<br>€ Other traditional method, specify
70.	How long have you been using the current method?	_____Years ______Months

G. Pregnancy and Birth History

71.	Are you currently pregnant?	€ Yes<br>€ No<br>€ Don't know
72.	How many months pregnant are you?	
For each birth since January 2009		
73.	What is the child's date of birth?	_______ Day _______ Month ______ Year
74.	Does the child have a birth certificate?	€ Yes<br>€ No
75.	Was it a single birth or multiple birth e.g. twins?	€ Single<br>€ Multiple
76.	What is the sex of the child?	€ Male<br>€ Female
77.	Is child still alive?	€ Yes<br>€ No
78.	How old was the child when he/she died?	

79.	At the time you became pregnant with baby, did you want to become pregnant then, did you want to wait until later, or did you not want to have any (more) children at all?	€ Then<br>€ Later<br>€ No more children
80.	During this pregnancy, did you see anyone for antenatal care?	€ Yes<br>€ No
81.	Why didn't you attend antenatal care?	€ Too expensive<br>€ Too far<br>€ Too busy<br>€ Self-treated<br>€ Was too early in pregnancy<br>€ Facility has poor structure<br>€ Facility poorly stocked<br>€ Poor staff attitude<br>€ Poor staff knowledge<br>€ Poor quality of care<br>€ Service not available<br>€ No transportation<br>€ Did not need/Not necessary<br>€ Inconvenient hours<br>€ Long waiting times<br>€ Prefer home care<br>€ Family didn't want me to go<br>€ Other (Specify):
82.	What would you say was the most important reason from above?	
83.	Where did you receive most of your antenatal care?	€ Your home<br>€ Other home<br>€ Govt. Hospital<br>€ (Enter PHC name from Q.3)<br>€ Other Primary Health Care Center (PHC)<br>€ Other public health facility<br>€ Private Hospital/clinic<br>€ Maternity/Nursing home<br>€ Other
84.	Who provided most of your care?	€ Doctor<br>€ Nurse/Midwife<br>€ Auxiliary midwife<br>€ Community Health Extension Worker (CHEW)<br>€ Traditional Birth Attendant (TBA)<br>€ No one<br>€ Others (specify)

85.	How many months pregnant were you when you first received antenatal care for this pregnancy?	
86.	How many times in total did you receive antenatal care during this pregnancy?	
87.	During those visits, was any of the following done during at least one visit?	€ Were you weighed<br>€ Was your height measured<br>€ Was your blood pressure measured<br>€ Did you give a urine sample<br>€ Did you give a blood sample<br>€ Did the provider palpate your tummy<br>€ Was your uterine height measured (this is when the provider measures your tummy using a measurement tape)<br>€ Did the health worker ask for your blood type<br>€ Did you receive advice on the diet during your pregnancy<br>€ Were you counseled on newborn care<br>€ Were you counseled on breastfeeding<br>€ Were you counseled and Tested for HIV<br>€ Were you told about the signs of pregnancy complications
88.	During this pregnancy, were you given an injection in the arm to prevent the baby from getting tetanus, that is, convulsions after birth?	€ Yes<br>€ No<br>€ Don't know
89.	During this pregnancy, did you take any drugs to keep you from getting malaria?	€ Yes<br>€ No<br>€ Don't know
90.	During this pregnancy, were you given or did you buy any iron tablets or iron syrup?	€ Yes<br>€ No<br>€ Don't know

91.	Did you experience any of the following during the pregnancy?	€ Severe headache<br>€ Blurred vision<br>€ Reduced/Absent fetal movement<br>€ High Blood pressure<br>€ Difficulty breathing<br>€ Loss of consciousness<br>€ Swollen hands and face<br>€ Convulsions<br>€ Excessive vaginal bleeding<br>€ Severe lower abdominal pain<br>€ High fever<br>€ Water break without labor<br>€ Don't know<br>€ Others, specify
92.	Where did you give birth?	€ Your home<br>€ Other home<br>€ Govt. Hospital<br>€ Enter PHC name from Q.3<br>€ Other Primary Health Care Center (PHC)<br>€ Other public health facility<br>€ Private Hospital/clinic<br>€ Maternity/Nursing home<br>€ Other
93.	Did you plan to give birth at home?	€ Yes<br>€ No
94.	What was your reason for not giving birth in a health facility?	€ Cost too much<br>€ Facility not open<br>€ Facility too far<br>€ No transportation<br>€ Don't trust facility/poor quality service<br>€ No female provider at facility<br>€ Husband/family did not allow<br>€ Not necessary<br>€ Not customary<br>€ Other (specify)
95.	Who assisted with the delivery of this pregnancy?	€ Doctor<br>€ Nurse/Midwife<br>€ Auxiliary nurse/midwife<br>€ Community Health Extension Worker (CHEW)<br>€ Traditional Birth Attendant (TBA)<br>€ No one<br>€ Others (specify)

96.	Did you plan to give birth at this place or did you go there because you experienced problems during labor/delivery?	€ Planned<br>€ Because of complications
97.	How did you go to the health facility?	€ Ambulance<br>€ Private car<br>€ Taxi/bus<br>€ Cart<br>€ Motorbike<br>€ Boat<br>€ On foot<br>€ Bicycle<br>€ Other (specify)
98.	How long did it take to reach the facility?	_______ HOURS _____ MINUTES
99.	How much did you pay for transportation to this facility? Include the cost of anyone who accompanied you	€
100.	In your opinion, how were the services in this facility?	€ Excellent<br>€ Good<br>€ Average<br>€ Poor
101.	Would you recommend this facility to your sister or friends?	€ Yes<br>€ No
102.	Can you tell me why you have ranked the services as Excellent/Good?	€ Provider always there<br>€ Provider is very competent<br>€ Facility always open<br>€ Staff respond to my questions<br>€ Facility always has necessary medicines<br>€ Not a long wait<br>€ Staff treat women with respect<br>€ Facility is clean, sanitary<br>€ Other<br>€ Don't know
103.	Can you tell me why you have ranked the services as Average/Poor?	€ Often health worker not there<br>€ Provider is not very competent<br>€ Often facility is closed<br>€ Staff do not answer my questions<br>€ Facility does not have necessary medicines<br>€ Long wait to be seen<br>€ Staff treat women poorly<br>€ Facility is dirty, unsanitary<br>€ Other<br>€ Don't know

104.	Was the doctor/nurse on site when you got to the facility?	€ Yes<br>€ No<br>€ Don't remember
105.	How much in total did you pay for the delivery including the cost of drugs?	N______
106.	How many nights did you spend in the health facility before you were allowed to go home?	
107.	Did you experience any health problems during labor and delivery?	€ Retained placenta (Placenta not delivered 30 minutes after baby)<br>€ Convulsions<br>€ Prolonged labor (>12hrs)<br>€ Loss of consciousness<br>€ Abnormal presentation e.g. breech, hand or feet coming first<br>€ Excessive vaginal bleeding<br>€ High fever<br>€ Others<br>€ No Problems
108.	When you experienced these symptoms, were you referred to a different health facility?	€ Yes<br>€ No
109.	To which facility were you referred?	€ Government General Hospital<br>€ Private Hospital/Clinic<br>€ Other (specify)
110.	Did you go to the referral facility?	€ Yes<br>€ No
111.	Why did you not go?	€ Cost too much<br>€ Facility not open<br>€ Too far<br>€ No transportation<br>€ Don't trust facility/poor quality service<br>€ No female provider at facility<br>€ Husband/family did not allow<br>€ Not necessary<br>€ Not customary<br>€ Other
112.	What was the most important reason?	

113.	How did you go to the referral facility?	€ Ambulance<br>€ Private car<br>€ Taxi/bus<br>€ Cart<br>€ Motorbike<br>€ Boat<br>€ On foot<br>€ Bicycle<br>€ Other
114.	How long did it take to reach the facility?	_______ HOURS ______ MINUTES
115.	How much did you pay for transportation to this facility? Include the cost of anyone who accompanied you	€
116.	Did you stay overnight in the referral facility?	€ Yes<br>€ No
117.	How many nights did you spend in the facility before you were allowed to go home?	
118.	How much in total did you pay for treatment including the cost of drugs?	N _________
119.	At what time of the day was baby born?	€
120.	Was baby born premature (i.e. before 9 months)?	€ Yes<br>€ No
121.	Was the baby born by Caesarean section (operation)?	€ Yes<br>€ No
122.	Was baby born by forceps or vacuum extraction?	€ Yes<br>€ No
123.	When baby was born, what was his/her relative size?	€ Very large<br>€ Larger than average<br>€ Average<br>€ Smaller than average<br>€ Very small<br>€ Don't know
124.	Was baby weighed at birth?	€ Yes<br>€ No
125.	How much did baby weigh?	_______ kg
126.	Did baby cry or breathe easily immediately after birth?	€ Yes<br>€ No

127.	How long after birth did you first put baby to the breast?	€ Hours ____<br>€ Days _____<br>€ Weeks _____<br>€ Don't remember<br>€ Never breastfed
128.	Did you give baby the first liquid that came from your breasts?	€ Yes<br>€ No
129.	Was baby exclusively breastfed for six months?	€ Yes<br>€ No<br>€ Baby is less than 6 months old<br>€ Don't remember
130.	Did baby experience any of the following within the 1st 7 days of being born?	€ Difficulty or fast breathing<br>€ Yellow skin/eye color (jaundice)<br>€ Poor sucking or feeding<br>€ Pus, bleeding, or discharge from around the umbilical cord<br>€ Skin lesions or blisters<br>€ Convulsions/spasms/rigidity<br>€ Lethargy/unconsciousness<br>€ Red or swollen eyes with pus<br>€ High fever<br>€ Other
131.	Did you go to a health facility for assistance?	€ Yes<br>€ No
132.	If yes, where did you receive most of the care?	€ Govt. Hospital<br>€ Enter PHC Name from Q.3<br>€ Other Primary Health Care Center (PHC)<br>€ Other public health facility<br>€ Private Hospital/clinic<br>€ Maternity/Nursing home<br>€ Other
133.	How much did you pay for transportation to this facility? Include the cost of anyone who accompanied you	€
134.	How much in total did you pay for treatment including the cost of drugs?	
135.	After baby was born, did any health care provider or a traditional birth attendant check on your health or the health of your newborn?	€ Yes<br>€ No

136.	How long after delivery did the first check take place?	€ Hours ____<br>€ Days _____<br>€ Weeks _____<br>€ Months _____<br>€ Don't remember
137.	Who checked on your health or your newborn health at that time?	€ Doctor<br>€ Nurse/Midwife<br>€ Auxiliary nurse/midwife<br>€ Community Health Extension Worker (CHEW)<br>€ Traditional Birth Attendant (TBA)<br>€ No one<br>€ Others (specify)
138.	Where did this first check take place?	€ Your home<br>€ Other home<br>€ Govt. Hospital<br>€ [Enter PHC Name from Q.3]<br>€ Other Primary Health Care Center (PHC)<br>€ Other public health facility<br>€ Private Hospital/clinic<br>€ Maternity/Nursing home<br>€ Other
139.	What was done by the health worker or others during the health check?	€ Examined my body<br>€ Checked breasts<br>€ Checked for heavy bleeding<br>€ Counseled on danger signs for newborn<br>€ Breastfeeding<br>€ Counseled on nutrition<br>€ Other (Specify)
140.	During the first 6 weeks after delivery did you experience any of the following health problems?	€ High fever<br>€ Lower abdominal pain<br>€ Foul smelling vaginal discharge<br>€ Severe bleeding<br>€ Convulsions<br>€ Loss of consciousness<br>€ Swollen hands/face<br>€ Difficulty breathing<br>€ Severe headache<br>€ Other (Specify)
141.	Did you go to a health facility for assistance?	€ Yes<br>€ No

142.	If yes, where did you receive most of the care?	€ Govt. Hospital<br>€ Enter PHC Name from Q.3<br>€ Other Primary Health Care Center (PHC)<br>€ Other public health facility<br>€ Private Hospital/clinic<br>€ Maternity/Nursing home<br>€ Other
143.	Did you stay overnight in the health facility?	€ Yes<br>€ No
144.	How many nights did you spend in the facility before you were allowed to go home?	
145.	How much in total did you pay for treatment including the cost of drugs?	

H. Knowledge and Perceptions

146.	Do you know of any health problems that can occur during pregnancy that could endanger the life of a pregnant woman?	€ Bleeding<br>€ Severe headache<br>€ Blurred vision<br>€ Convulsions<br>€ Swollen hands/face<br>€ High fever<br>€ Loss of consciousness<br>€ Difficulty breathing<br>€ Severe weakness<br>€ Severe abdominal pain<br>€ Accelerated/ reduced fetal movement<br>€ Water breaks without labor<br>€ Other (Specify):<br>€ Don't know any
147.	Do you know of any health problems that can occur during labor and childbirth that could endanger the life of a pregnant woman?	€ Severe bleeding<br>€ Severe headache<br>€ Convulsions<br>€ High fever<br>€ Loss of consciousness<br>€ Prolonged labor (>12 hours)<br>€ Retained placenta (placenta not delivered 30 minutes after baby)<br>€ Other (Specify):<br>€ Don't know any
148.	Do you think there are any good reasons for a woman to receive antenatal care?	€ Yes<br>€ No
149.	What are those reasons?	€ Prevent malaria<br>€ Health education<br>€ Get medicine<br>€ Prevent anemia in pregnancy<br>€ To see doctor<br>€ Early detection and management of complication during pregnancy<br>€ Other (Specify)<br>€ Don't know any reasons

150.	Where did you hear/learn about these reasons?	€ Husband<br>€ Mother-in-law<br>€ Other relative<br>€ Friend<br>€ Radio<br>€ Television<br>€ Newspaper<br>€ Health care worker<br>€ Church or Mosque<br>€ Community meeting<br>€ Town hall<br>€ Community theatre<br>€ SMS on cell phone<br>€ Internet<br>€ Town crier
151.	Do you agree/disagree with the following statement:<br>"Every woman should give birth in a health facility"	€ Strongly Agree<br>€ Agree<br>€ Neither Agree nor Disagree<br>€ Disagree<br>€ Strongly Disagree
Now I would like to ask you some questions about _____ [Enter PHC name from Q.3]		
152.	In your opinion, how are the services in this facility?	€ Excellent<br>€ Good<br>€ Average<br>€ Poor<br>€ Don't know
153.	Would you recommend this facility to your sister or friends?	€ Yes<br>€ No
154.	Can you tell me why you have ranked the services as Excellent/Good?	€ Provider always there<br>€ Provider is very competent<br>€ Facility always open<br>€ Staff respond to my questions<br>€ Facility always has necessary medicines<br>€ Not a long wait<br>€ Staff treat women with respect<br>€ Facility is clean, sanitary<br>€ Other<br>€ Don't know

155.	Can you tell me why you have ranked the services as Average/Poor?	€ Often health worker not there<br>€ Provider is not very competent<br>€ Often facility is closed<br>€ Staff do not answer my questions<br>€ Facility does not have necessary medicines<br>€ Long wait to be seen<br>€ Staff treat women poorly<br>€ Facility is dirty, unsanitary<br>€ Other<br>€ Don't know
156.	Have you ever heard about the Midwives Service Scheme (MSS) Program?	€ Yes<br>€ No
157.	How did you first hear about it?	€ Radio/TV<br>€ Newspaper/magazine<br>€ Posters/pamphlets<br>€ Friends/neighbors<br>€ Health worker<br>€ Ward development committee<br>€ Other
158.	Is there an MSS facility in your community?	€ Yes<br>€ No<br>€ Don't Know
159.	How would you rate the impact the MSS has had on access to midwives in your community?	€ Large positive impact<br>€ Moderate positive impact<br>€ Small positive impact<br>€ No impact<br>€ Negative impact
160.	How would you rate the impact the MSS has had on quality of maternal health services in your community?	€ Large positive impact<br>€ Moderate positive impact<br>€ Small positive impact<br>€ No impact<br>€ Negative impact
161.	How would you rate the impact the MSS has had on health outcomes in your community?	€ Large positive impact<br>€ Moderate positive impact<br>€ Small positive impact<br>€ No impact<br>€ Negative impact

I. Immunization History

162.	Do you have a vaccination card where NAME'S vaccinations are written down?	€ Yes, seen<br>€ Yes, not seen<br>€ No
163.	BCG	Day/Month/Year
164.	OPV0	Day/Month/Year
165.	OPV1	Day/Month/Year
166.	OPV2	Day/Month/Year
167.	OPV3	Day/Month/Year
168.	DPT1	Day/Month/Year
169.	DPT2	Day/Month/Year
170.	DPT3	Day/Month/Year
171.	Measles	Day/Month/Year
172.	Vitamin A 1st dose	Day/Month/Year
173.	Vitamin 2nd dose	Day/Month/Year
174.	Has NAME received any vaccination or Vitamin A not recorded on this card, including vaccination given on a national immunization day or child health week?	€ Yes, Date:<br>€ No
175.	Did NAME ever receive any vaccination to prevent him/her from getting disease including vaccine received on national immunization day or child health week?	€ Yes<br>€ No
176.	Did NAME receive a BCG vaccination against tuberculosis that is an injection in the forearm that usually causes a scar?	€ Yes<br>€ No
177.	Did NAME receive a polio vaccine—that is, drops in the mouth?	€ Yes<br>€ No
178.	When did he/she receive the polio vaccine the first time?	€ Just after birth<br>€ Later
179.	How many times was the polio vaccine given?	
180.	Did NAME receive a DPT vaccine—that is, an injection in the thigh usually given at the same time as the polio vaccine?	€ Yes<br>€ No
181.	How many times was the DPT vaccine given?	

182.	Did NAME receive a measles injection or an MMR injection—that is, an injection in the arm at the age of 9 months or older to prevent from getting measles?	€ Yes<br>€ No
183.	Did NAME receive this measles vaccine before he/she turned one year old, or after?	€ Before<br>€ After
184.	Did NAME ever receive a vitamin A supplement during a national immunization campaign or child health week?	€ Yes<br>€ No
